# Combating multidrug resistance and metastasis of breast cancer by endoplasmic reticulum stress and cell-nucleus penetration enhanced immunochemotherapy

**DOI:** 10.7150/thno.71693

**Published:** 2022-03-21

**Authors:** Weixi Jiang, Li Chen, Xun Guo, Chen Cheng, Yuanli Luo, Jingxue Wang, Junrui Wang, Yang Liu, Yang Cao, Pan Li, Zhigang Wang, Haitao Ran, Zhiyi Zhou, Jianli Ren

**Affiliations:** 1 Department of Ultrasound and Chongqing Key Laboratory of Ultrasound Molecular Imaging, the Second Affiliated Hospital of Chongqing Medical University, Chongqing 400010, P. R. China; 2 Department of Intensive Care Unit, the Second Affiliated Hospital of Chongqing Medical University, Chongqing 400010, P. R. China; 3 Department of Radiology, the Second Affiliated Hospital of Chongqing Medical University, Chongqing 400010, P. R. China; 4 Department of Gynecology and Obstetrics, the Second Affiliated Hospital of Chongqing Medical University, Chongqing 400010, P. R. China; 5 Department of General practice of Chongqing General Hospital, University of Chinese Academy of Sciences, Chongqing 401147, P. R. China

**Keywords:** ER stress, cell-nucleus penetration, immunogenic cell death, ATP-responsive drug release, multidrug resistance

## Abstract

**Rationale:** Multidrug resistance (MDR) and metastasis of breast cancer remain major hurdles in clinical anticancer therapy. The unsatisfactory outcome is largely due to insufficient cytotoxicity of chemotherapeutic agents and limited immunogenic cell death (ICD). On the other hand, efflux proteins, especially P-glycoprotein (P-gp), can recognize and promote the efflux of drugs from tumor cells.

**Methods:** In this study, silver nanoparticles (Ag NPs) and peptide- functionalized doxorubicin (^P^DOX) were used to prepare a theranostic nanocomposite (Ag-TF@^P^DOX), which induced organelle-mediated immunochemotherapy and drug efflux protein inhibition in drug-resistant breast cancer cells (MCF-7/ADR) via a strategy based on endoplasmic reticulum (ER) stress and cell-nucleus penetration.

**Results:** The silver nanoparticle-triggered persistent activation of ER stress synergizes with chemotherapy to enhance cytotoxicity and stimulate the ICD effect. It has the potential to enhance chemosensitivity by downregulating of P-gp expression due to the increased production of ATP-consuming chaperones. In addition, the novel peptide (CB5005), which not only penetrates the cell membrane but also has a nuclear localization sequence, is conjugated to DOX to improve both cellular internalization and intranuclear accumulation. Moreover, surface TA-Fe^3+^ engineering endows the nanocomposite with ATP-responsive disassembly and ATP depletion properties to improve biocompatibility and decrease ATP-dependent drug efflux. Ag-TF@^P^DOX has potential as a dual-mode (PAI/MRI) contrast-enhanced agent for realizing theranostic guidance.

**Conclusion:** This theranostic nanocomposite greatly restricts the growth of drug-resistant breast tumors and activates a strong immune response as well, providing an opportunity for the development of therapeutics that reverse tumor MDR and metastasis at the subcellular level.

## Introduction

In the last decade, although extensive efforts have been devoted to reversing multidrug resistance (MDR) by employing various nanotechnology-based strategies involving enhanced drug accumulation based on the targeting of tumors 1, 2, the prevention of drug-targeted pathways deactivation 3 or the combination of multiple antitumor therapies 4, 5, however, MDR remains an important factor that limits the efficacy of chemotherapy 6-8. As one of the best characterized classical chemotherapeutic agents, doxorubicin (DOX) has been widely utilized in the clinical practice for cancer treatment. Moreover, DOX-induced immunogenic chemotherapy can initiate immunogenic cell death (ICD), which is considered as a distinctive cell death program that can elicit an immune response by exposing death-associated molecular patterns (DAMPs) on the surface of dying cancer cells to induce antitumor immune effects 9-11. Unfortunately, chemotherapy alone cannot to provoke profound ICD to a satisfactory extent, largely due to the immunosuppressive tumor microenvironment 11. Worse still, because efflux proteins, especially P-glycoprotein (P-gp), are overexpressed on the plasma membranes of drug-resistant breast cancer cells, DOX that reaches the cytoplasm is rapidly pumped out of the cell, thereby reducing the cellular drug concentration 12-14. This may impair the cytotoxicity of chemotherapeutic agents and fail to produce a sufficient number of dying apoptotic cells that release DAMPs, thus further restricting the immune response at the same time. Therefore, we realized that to overcome the MDR and metastasis of breast cancer, a highly efficient nanocomposite that is capable of not only synergistically enhancing DOX-induced chemotherapy and ICD but also of downregulating the intracellular expression of P-gp, needs to be developed.

It is noteworthy that designing versatile nanoagents based on subcellular-level pathogenesis has become a new pattern and trend in drug discovery 15, 16. Recently, an organelle-mediated cancer therapy approach involving the endoplasmic reticulum (ER), the largest structure in cells, has also recently attracted researchers' attention 17-19. It has been demonstrated that disturbing the homeostasis of ER would lead to ER stress, which directly influences cell survival 20, 21. Notably, despite that a mild or transient ER stress was believed to promote cancer cell homeostasis initially under harsh environmental conditions and confer resistance to chemotherapy 22, 23, some recent studies found that uncontrolled ER stress induced by silver nanoparticles (Ag NPs) 24, 25 may stimulate apoptosis by mediated transcription factor CCAAT/enhancer-binding protein homologous protein (CHOP), and inhibit P-gp expression due to increased production of ATP-consuming chaperones, rendering MDR cells more vulnerable to anticancer drugs 26, 27. More importantly, cancer cells under persistent ER stress during the early phase of apoptosis release an abundance of DAMPs, such as calreticulin (CRT) and high mobility group box 1 (HMGB1), which send the “eat me” signal to Toll-like receptor 4 on the surface of dendritic cells (DCs), thus promoting DC maturation. DCs then present antigens to stimulate the proliferation of CD8^+^ T cells, which can differentiate into cytotoxic T lymphocytes (CTLs) and generate inflammatory cytokines to attack cancer cells 9, 28. Therefore, the combination of chemotherapy and irreversible Ag NP-mediated ER stress is believed to have a synergistic, two-pronged effect of improving tumor chemosensitivity and ICD effect 29-31. On the other hand, the efficacy of DOX is highly dependent on nuclear access because it induces cell death by interacting with the DNA helix or related enzymes 32. Although, DOX can enter the cell nucleus by passive diffusion, we found that the efficiency of nuclear entry through this method is substantially limited in MDR cancer cells probably due to the nature of their drug resistance 33, 34. Recently, a novel dual-functional cell nucleus-penetrating peptide (CPP), named CB5005, which consists of a cell membrane-permeable sequence cascaded with a nuclear localization sequence (NLS), has come into favor with researchers due to its applicability in biomedicine. With the assistance of this peptide, drugs can effectively penetrate the membranes of cancer cells, bypass the subcellular barriers and concentrate around the nucleus, thus maximizing their therapeutic efficacy 35. Therefore, we aimed to construct a CPP-functionalized anticancer drug to enhance the active nuclear targeting and cell membrane penetration of DOX, thereby reversing MDR. Ultimately, for the simultaneous delivery of Ag NPs and peptide-functionalized DOX (^P^DOX), the metal-phenolic network, tannic acid-iron^3+^ (TA-Fe^3+^), was chosen as the best nanocarrier for the following reasons: (1) The excellent interfacial cohesion and accompanying supramolecular organization of TA-Fe^3+^ allows the delivery of nanoscale ^P^DOX particles and tactful immobilization of Ag NPs in the nanocomposite due to the polyphenol structure of TA 36-39; (2) Fe^3+^ shows a strong binding affinity for ATP through metal ion-triphosphate coordination, which could disassemble TA-Fe^3+^ networks to release drugs and synergistically deplete cellular energy with ER stress to impair ATP-dependent drug efflux and thus reverse MDR 17, 40, and (3) TA-Fe^3+^ is an excellent candidate for magnetic resonance imaging (MRI) and photoacoustic imaging (PAI), providing theranostic guidance and visual monitoring during treatment to obtain comprehensive and accurate information 41, 42.

**Scheme 1:**
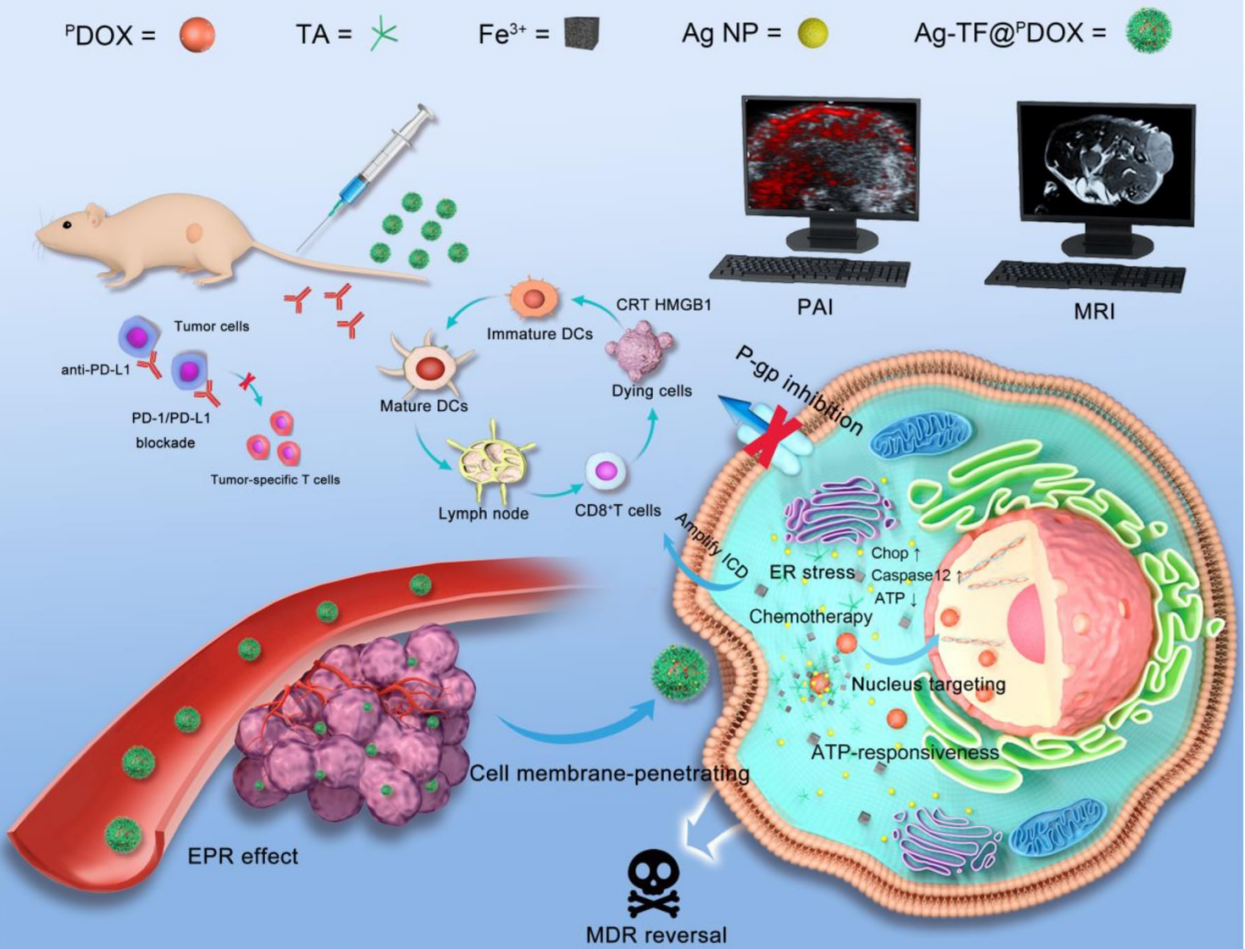
Schematic illustration of organelle-mediated immunochemotherapy for combating MDR and metastasis of breast cancer. First, the Ag-TF@^P^DOX nanocomposite was intravenously injected and then delivered to drug-resistant MCF-7 tumors via the EPR effect under PAI and MRI guidance. Then, TA-Fe^3+^ depleted abundant ATP in the tumor microenvironment and disassembled, releasing the Ag NPs and CB5005-functionalized DOX. The Ag NPs induced uncontrolled ER stress leading to a two-pronged effect, which not only triggered apoptotic signaling but also downregulated P-gp expression. DOX possessed cell membrane-penetrating and nucleus-targeting capabilities was further delivered to the nucleus, thus maximizing therapeutic efficacy for combating drug resistance. Moreover, an abundance of DAMPs synergistically provoked by ER stress and nucleus-targeting chemotherapy were exposed. Then, DAMPs were presented by DCs to trigger an immune response in combination with PD-1/PD-L1 blockade.

Upon passive accumulation of the versatile nanocomposite in tumors via the enhanced permeability and retention (EPR) effect, the release of ^P^DOX and Ag NPs was quickly triggered by TA-Fe^3+^ dissolution under the condition of high ATP levels (1×10^-3^-10×10^-3^ M) in tumor cells 43. Next, the amplified apoptotic signal and immune response were synergistically activated by ER stress and nucleus-targeting chemotherapy. ER stress and ATP consumption doubly inhibited P-gp expression to combat tumor drug resistance. Additionally, anti-programmed cell death ligand-1/ligand-L1 (PD-1/PD-L1) blockade, which can relieve immunosuppression and enhance nanocomposite-based immunochemotherapeutic efficacy, was applied *in vivo* to suppress the growth of both primary breast tumor growth and the progression to metastasis **(Scheme 1)** 44-46.

## Results and Discussion

### Preparation and Characterization of the Ag-TF@^P^DOX Nanocomposites

**Scheme 2:**
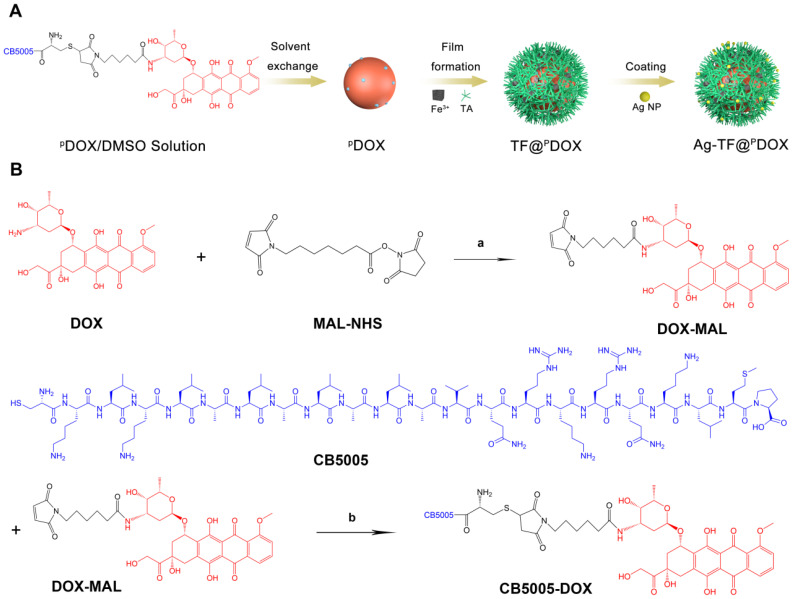
A) Schematic on the synthetic procedure of Ag-TF@^P^DOX. B) The synthetic routes of CB5005 conjugated DOX and the related chemical structures. (a) TEA, 37 °C, 1 h; (b) 37 °C, 6 h.

**Figure 1:**
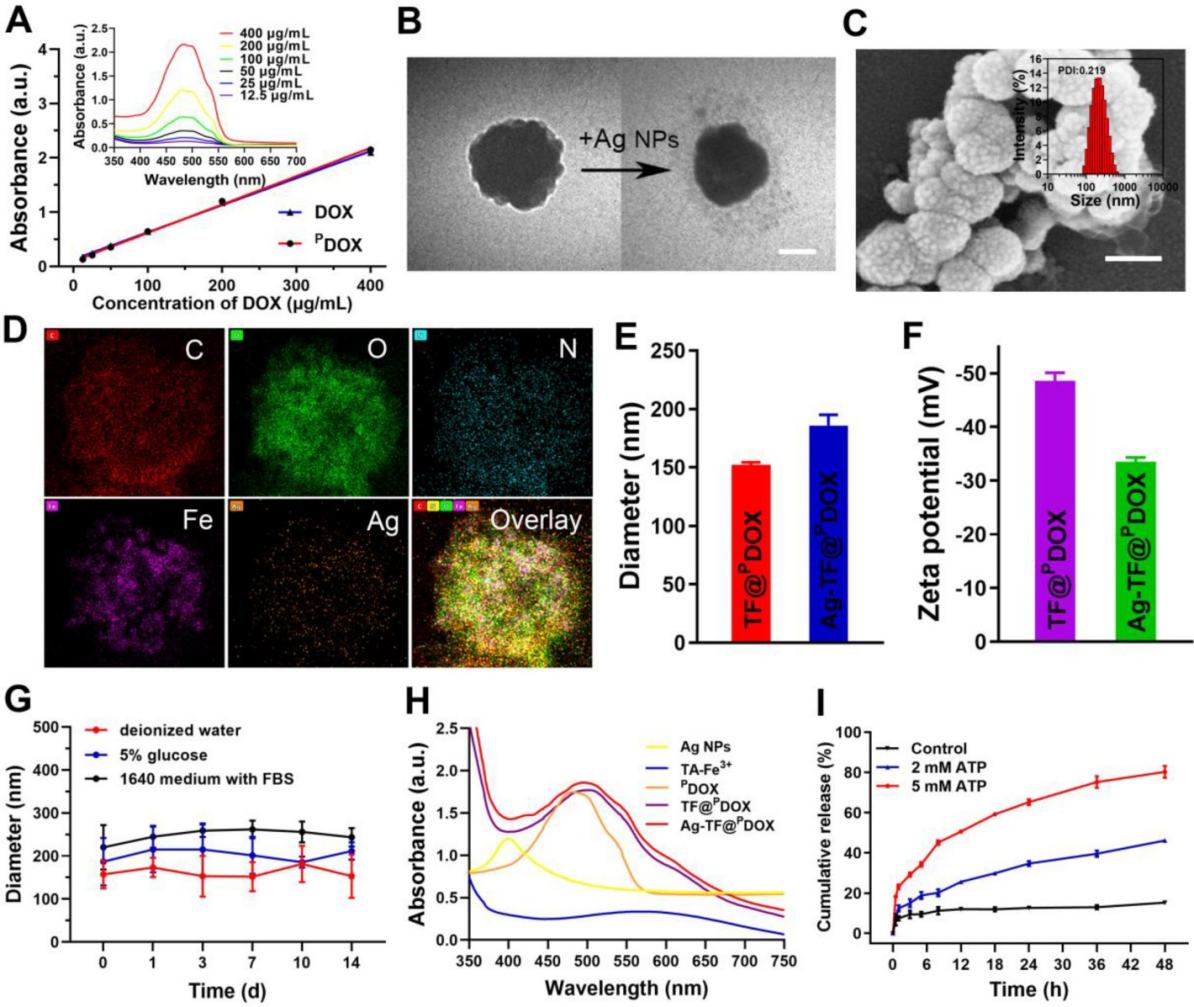
Characterizations of Ag-TF@^P^DOX nanocomposite. A) Ultraviolet absorbance of DOX and ^P^DOX at different concentrations (inset: UV spectrum of ^P^DOX at different concentrations). B) TEM images of nanocomposite before and after coating Ag NPs, the scale bar is 0.1 μm. C) An SEM image of Ag-TF@^P^DOX, the scale bar is 0.2 μm (inset: size distribution of Ag-TF@^P^DOX). D) Elemental line-scan mapping of Ag-TF@^P^DOX. E) Diameters and F) Zeta potentials of nanocomposite before and after coating Ag NPs, n = 3. G) Size changes of Ag-TF@^P^DOX during 14-day observation. H) UV spectrum of Ag NPs, TA-Fe3^+^, ^P^DOX, TF@^P^DOX, and Ag-TF@^P^DOX. I) ATP-responsive drug release under different ATP concentrations (0, 2 and 5 mM) at various time intervals, n = 3.

The preparation process of the versatile nanocomposite is illustrated in **Scheme 2A**. The molecular weight of this dual-functional peptide was confirmed by mass spectrometry (MS) and found to be comparable to that of the desired amino acid sequence (2493.22 Da) **(Figure S1)**. High-performance liquid chromatography (HPLC) showed that the purity of polypeptide was up to 98%, indicating the sound synthesis of CB5005 **(Figure S2)**. To construct a nucleus-targeting chemotherapeutic, DOX was initially attached to a rationally designed NLS sequence that was also capable of cell membrane penetration via a maleimide-thiol click reaction between cysteine residues on polypeptide and DOX-MAL **(Scheme 2B)**. MS results revealed that the molecular weights of DOX were increased from 544.18 to 759.30 Da after reacting with 6-Maleimidocaproic acid, indicating that DOX was modified by MAL group **(Figure S3)**. The proton nuclear magnetic resonance spectroscopy (^1^HNMR) spectrum of CB5005 fitted to the spectrum of DOX displayed peaks from 7.6 to 7.9 ppm, which were attributed to the hydrogen proton peak of the benzene ring, indicating the successful conjugation of CB5005 with DOX **(Figure S4)**. The molecular weights of the nanocomposites after conjugation were also characterized using MS, as depicted in **Figure S5**. Moreover, the disappearance of the characteristic peaks of CB5005 and DOX in the HPLC trace also validated the construction of ^P^DOX **(Figure S6)**. Next, we compared the ultraviolet (UV) absorbance of ^P^DOX at doses equivalent to those of commercially available DOX at various concentrations. High conformity was observed, as depicted in **Figure 1A**, suggesting that DOX features were preserved during the attachment process. Subsequently, TA and Fe^3+^ aqueous solutions were sequentially added to a ^P^DOX/DMSO solution with continuous sonication, vortexed and subjected to pH neutralization. TA can coordinate with Fe^3+^ to form a three-dimensional network film on the surface of the ^P^DOX core, producing nanoscale particles (designated TF@^P^DOX) within seconds. Notably, the mass ratio of the feeding drug and TA-Fe^3+^ may affect the encapsulation efficiency of the nanocomposite; therefore, ^P^DOX and TA-Fe^3+^ with different masses were initially incorporated to investigate the optimal feeding ratio. Interestingly, we found that even though an increase in TA-Fe^3+^ improved the loading capability of the nanocomposite, precipitation was evident, and the particles appeared larger, as demonstrated in **Table S1**; hence, we performed HPLC and dynamic light scattering (DLS) to measure the encapsulation efficiency and size distribution of the particles and found that a ^P^DOX:TA-Fe^3+^ feeding ratio of 1:5 was optimal. Furthermore, considering the highly adhesive nature of the polyphenol-structured TA network, small Ag NPs (10 nm) were coated onto TF@^P^DOX, which endowed this nanocomposite (designated Ag-TF@^P^DOX) with greater ER stress induction capacity 47. Transmission electron microscopy (TEM) revealed that TF@^P^DOX had a spherical morphology with nanoscale dimensions. After addition of Ag NPs and stirring, numerous Ag NPs clustered around the surface of the nanosystem **(Figure 1B and Figure S7)**. Scanning electron microscopy (SEM) also revealed that the surface of Ag-TF@^P^DOX was rough due to the adsorption of the Ag NPs **(Figure 1C)**. The elaborate design was further verified by elemental mapping and X-ray photoelectron spectroscopy (XPS), which revealed a clear distribution of Fe and Ag elements in the nanocomposite shell** (Figure 1D and Figure S8)**. The size distributions of the nanocomposite before and after the addition of Ag NPs were assessed using DLS, which revealed that the nanocomposite had a narrow distribution (PDI: 0.215) with a hydrodynamic diameter change from 152.2 nm to 185.9 nm **(Figure 1E)**. The zeta potential of TF@^P^DOX after Ag NP adherence increased from -48.6 mV to -33.6 mV, as shown in **Figure 1F**. In addition, nonsignificant diameter changes of Ag-TF@^P^DOX in various media such as deionized water, 1640 culture medium containing 10% FBS, and 5% glucose solution were observed during a 14-day storage period, indicating good stability of the nanocomposite **(Figure 1G)**. Next, UV spectroscopy revealed that both TF@^P^DOX and Ag-TF@^P^DOX had an absorption peak at 495 nm, which was slightly redshifted with respect to the characteristic absorption peak of ^P^DOX (480 nm), as shown in **Figure 1H**, suggesting the successful loading of CPP-conjugated antitumor drugs. In addition, the ^P^DOX encapsulation efficiency was found to be approximately 80.4% according to the standard curve of ^P^DOX obtained by UV spectroscopy **(Figure S9)**, which was similar to the HPLC results **(Figure S10)**. The encapsulation efficiencies of Fe^3+^ and Ag NPs were determined to be 39.0% and 41.6%, respectively, by using inductively coupled plasma-mass spectrometry (ICP-MS). To verify the ATP-triggered disassembly behavior of Ag-TF@^P^DOX, the amount of ^P^DOX released at different ATP concentrations and time points was quantified. As shown in **Figure 1I**, the release efficiency of the nanocomposite was relatively low in the absence of ATP, but it increased rapidly over time in groups containing ATP. Approximately 80.2% of the encapsulated ^P^DOX was released from Ag-TF@^P^DOX after 48 h in the presence of a high concentration of ATP (5 mM). The color of the buffer solutions and the amount of residual drugs in the dialysis bags also validated the release rate **(Figure S11)**. The novel ATP-responsive nanocomposite was attributed to the binding affinity of Fe^3+^ for ATP, which leads to the “burst release” of the drug in the presence of abundant ATP in tumor cells.

### Endoplasmic Reticulum Stress

**Figure 2:**
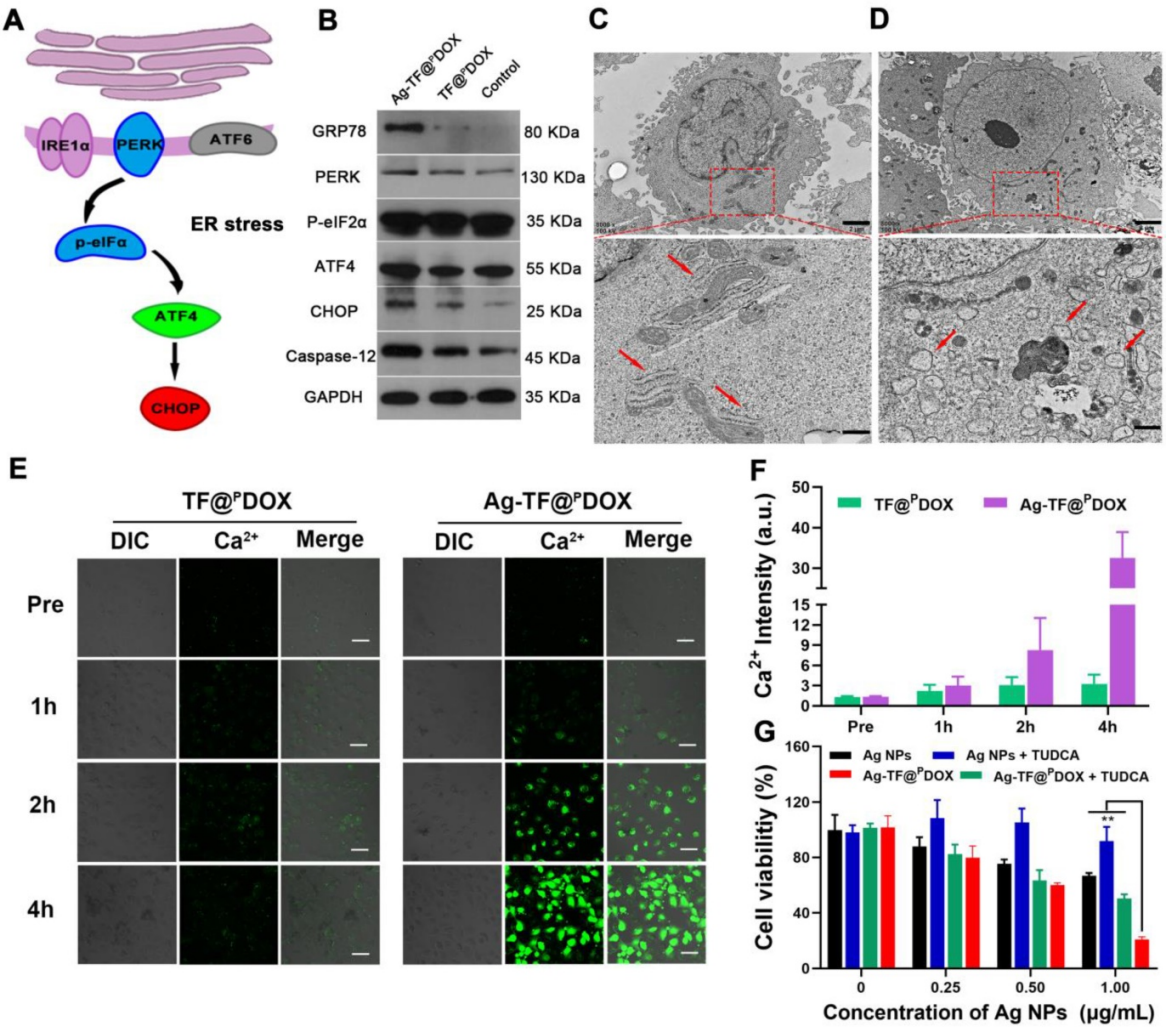
ER stress-mediated cell death**.** A) Schematic illustration on ER stress signaling pathway. B) Western blotting result of ER stress response markers in MCF-7/ADR after treating with culture medium, TF@^P^DOX, and Ag-TF@^P^DOX. Bio-TEM images of MCF-7/ADR cells C) before and D) after incubating with Ag-TF@^P^DOX for 6 h. The scale bars are 2 μm. The red squares in the images reveal the sites of endoplasmic reticulum in MCF-7/ADR cells. The scale bars are 0.8 μm. The red arrows indicate endoplasmic reticulum in physical or stress conditions. E) CLSM images and F) relative fluorescence intensity of Ca^2+^ level in the cytoplasm of MCF-7/ADR as detected by Fluo-8 after incubation with TF@^P^DOX, and Ag-TF@^P^DOX for 0, 1, 2, 4 h. The scale bars are 50 μm. G) Cell viabilities in present or absent of TUDCA after incubation with different concentrations of Ag NPs or Ag-TF@^P^DOX for 24 h, n = 3.

The ER has been revealed to control the synthesis, folding, and assembly of proteins and other biological macromolecules, and it plays an important role in cell survival as well 48. Several studies have reported that Ag NPs can induce tumor cell apoptosis via modulation of the ER stress pathway, and therefore, Ag NPs were included in the nanosystem **(Figure 2A)**. The western blotting results showed that the expression levels of the dominant ER stress signaling proteins GRP78 and PERK were increased in MCF-7/ADR cells after Ag NPs addition, compared with those in the TF@^P^DOX and control groups. Other hallmarks of the ER stress signaling pathway, including the activation of ATF4, CHOP, and Caspase-12, key indicators of the proapoptotic process, were also detected **(Figure 2B)**. These results demonstrated that the Ag-TF@^P^DOX nanocomposite is likely to induce C/EBP-homologous protein-mediated apoptosis via the PERK-eIF2α-ATF4 signaling pathway. Next, we observed morphological changes in the ER after the incubation of cells with Ag-TF@^P^DOX, as determined by Bio-TEM images. The ER displayed a series of lamellar and tubular cavities composed of membranes under physiological conditions (**Figure 2C**). When stimulated, some of the ER lost its physiological form, showing noticeable swelling and dilation after incubation with Ag-TF@^P^DOX, as illustrated in **Figure 2D and Figure S12.** It has been reported that ER stress can disrupt cellular calcium stores, leading to Ca^2+^ release into the cytoplasm; therefore, measurement of the intracellular Ca^2+^ level may provide indirect insight into the occurrence of ER stress. MCF-7/ADR cells treated with Ag-TF@^P^DOX and TF@^P^DOX for 1, 2, and 4 h were stained with Fluo-8, a cell-permeable Ca^2+^ sensor that binds free Ca^2+^ in the cytoplasm but does not bind Ca^2+^ in the ER. As shown in **Figure 2E and 2F,** the intensity of the Ca^2+^-triggered fluorescent signal increased noticeably in the Ag-TF@^P^DOX-treated cells over time, indicating that a high intracellular level of free Ca^2+^ was released from the ER; however, no significant fluorescence intensity change was observed in the TF@^P^DOX-treated cells. In addition, sodium tauroursodeoxycholate (TUDCA), a classic ER stress suppressor that reduces ERK phosphorylation, was used in this study. Interestingly, the cytotoxicity of both the Ag NPs and Ag-TF@^P^DOX were decreased in cells in the presence of TUDCA compared with that in cells not treated with TUDCA, suggesting again that Ag NP-related cell death was activated by ER stress **(Figure 2G)**.

### ATP Depletion and P-gp Inhibition

**Figure 3:**
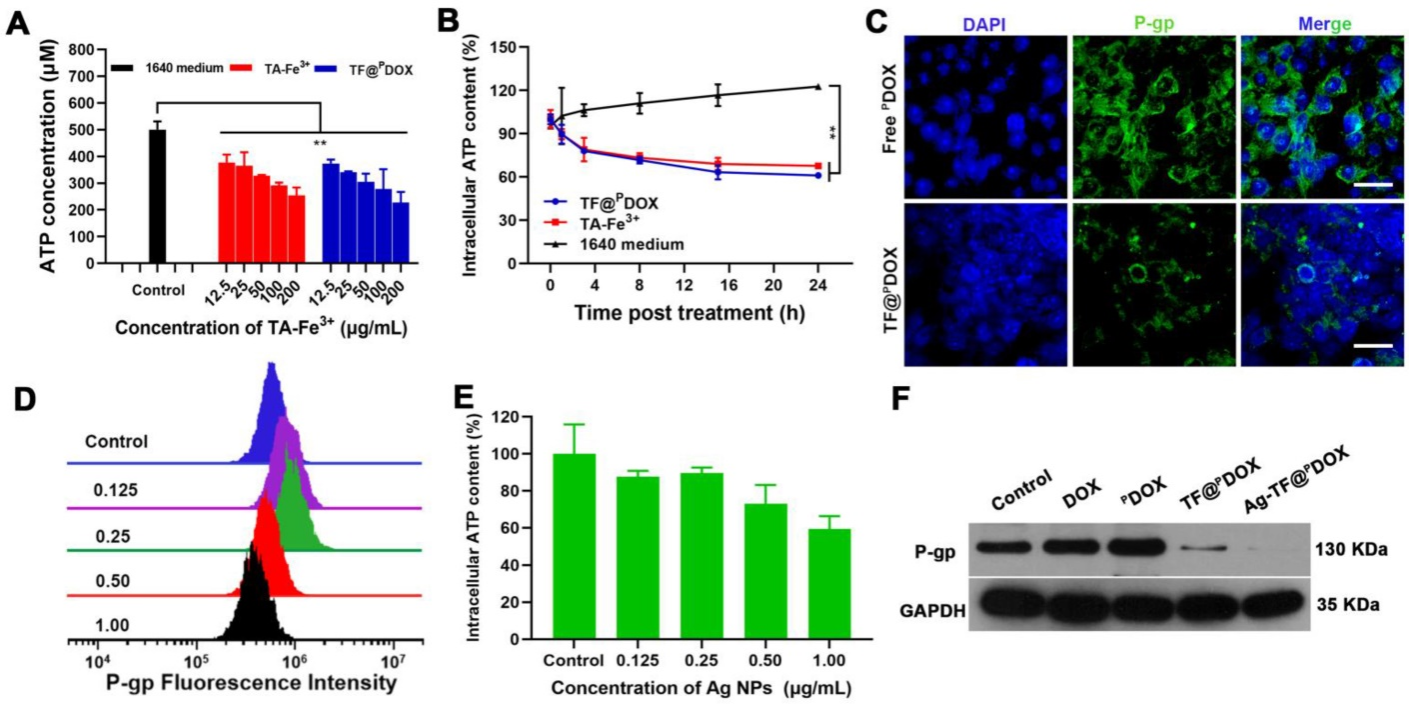
ATP depletion efficiency and P-gp inhibiting property of nanocomposite. A) Residual ATP content in solution determined by ATP assay kit after treating with 1640 medium, TA-Fe^3+^, and TF@^P^DOX, n = 3. B) Intracellular ATP content in MCF-7/ADR cells after different treatments at various time intervals, n = 3. C) Confocal images of MCF-7/ADR cells after 24 h incubation with free ^P^DOX or TF@^P^DOX, respectively. The P-gp in membrane and cytoplasm are stained with FITC and nuclei are stained with DAPI. The scale bars are 50 μm. D) Flow cytometry analysis of intracellular P-gp expression after treating with Ag NPs at various concentrations. E) Intracellular ATP content in MCF-7/ADR cells after treating with Ag NPs, n = 3. F) Western blotting results of P-gp expression in MCF-7/ADR cells after incubation with different agents.

Considering that the TA-Fe^3+^-covered nanocomposite can deplete the high level of ATP in tumor cells and thus suppress ATP-dependent drug efflux, we first measured the change in ATP content in a solution after mixing Roswell Park Memorial Institute (RPMI)-1640 culture medium with TA-Fe^3+^ and TF@^P^DOX at various concentrations for 24 h. As shown in **Figure 3A**, the ATP consumption efficiency was similar in the TF@^P^DOX and TA-Fe^3+^ solutions containing equivalent doses of TA-Fe^3+^, which suggested that the nanosystem retained the potent ATP-binding capability of Fe^3+^. Next, the intracellular ATP level in MCF-7/ADR cells was measured after the cells were coincubated with TF@^P^DOX, TA-Fe^3+^ and 1640 culture medium for various times. The results revealed that the cancer cells in either the TF@^P^DOX or TA-Fe^3+^ groups showed notably decreased intracellular ATP levels compared with those in culture medium alone, showing that the nanosystem exhibited a favorable ATP consumption performance **(Figure 3B)**. Given this fact, we evaluated the expression of P-gp as driven by ATP because P-gp plays a crucial role in MDR. As depicted in **Figure 3C**, a distinct green signal was mainly distributed in the cell plasma membrane of MCF-7/ADR cells treated with ^P^DOX, demonstrating the overexpression of drug efflux pumps in MDR cells. However, the fluorescence intensity was obviously impaired when the cells were treated with TF@^P^DOX, possibly because P-gp expression was inhibited due to a decrease in the amount of ATP. These studies demonstrated that the TA-Fe^3+^-coated nanosystem may hold great potential for reversing P-gp-mediated tumor resistance by depleting ATP in tumor cells. Although several notable studies have reported that mild ER stress generally benefits cellular homeostasis, which might facilitate drug resistance, some findings demonstrated that the effect of sustained and severe ER stress may result in a reverse effect, favoring cell death instead of survival. This suggests that uncontrolled activation of ER stress has the potential to be a strategy for enhancing chemosensitivity. To verify this, a variety of Ag NP concentration gradients that can stimulate ER stress to different degrees were initially prepared to investigate the Ag NP effect on intracellular P-gp expression. Flow cytometry analysis showed that, although exposure to low-dose Ag NPs only slightly increased P-gp levels, P-gp expression was downregulated markedly when the concentration of Ag NPs was greater than 0.5 μg/mL **(Figure 3D)**. It was reported that ER stress causes increased production of ATP-consuming chaperones, resulting in ATP loss. To investigate the mechanism of P-gp inhibition initiated by ER stress, intracellular ATP levels after treatment with Ag NPs were studied. Based on the results **(Figure 3E)**, a high concentration of Ag NPs markedly reduced the ATP concentration in MDR cells. Furthermore, different drug formulations were used to determine the degrees of influence on intracellular P-gp expression. As expected, both western blotting and flow cytometry results revealed that TF@^P^DOX and Ag-TF@^P^DOX reduced intracellular P-gp expression, and the Ag-TF@^P^DOX had the strongest ability to inhibit P-gp, probably because ATP depletion and ER stress exerted a synergistic effect **(Figures 3F and S13)**. These results indicated that TA-Fe^3+^ engineering and ER stress are capable of simultaneously enhancing chemotherapy sensitivity to combat MDR.

### ER Stress and Chemotherapy Synergistically Amplified ICD

**Figure 4:**
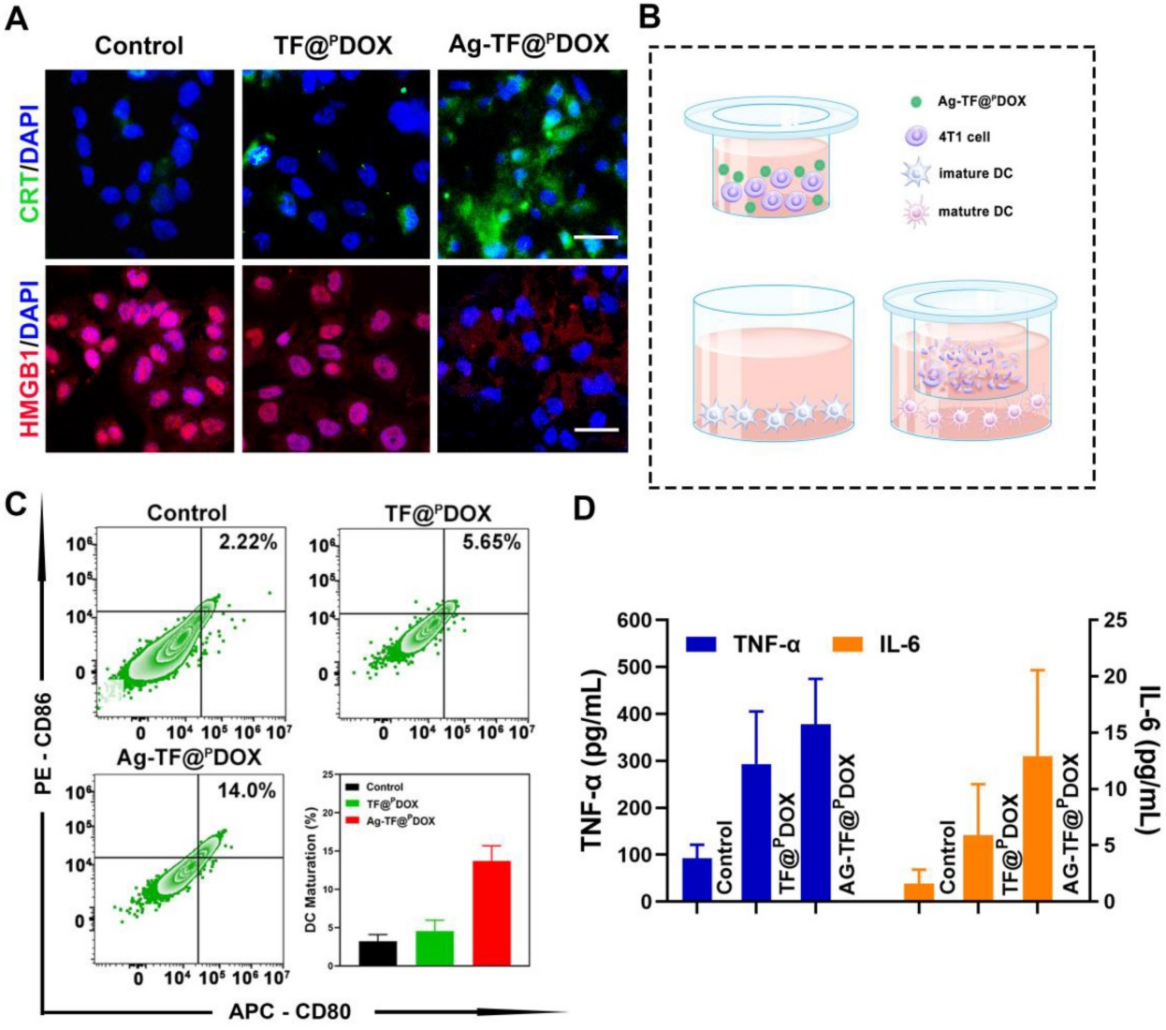
Characterization of the ICD effect. A) Fluorescent images of intracellular CRT and HGMB1 expression of MCF-7/ADR cells after treating with culture medium, TF@^P^DOX, and Ag-TF@^P^DOX for 4 h. CRT is stained with FITC, HMGB1 is stained with Dylight 649, and nuclei are stained with DAPI. The scale bars are 50 μm. B) Schematic on the coincubation procedure of Transwell assay. C) Flow cytometry and corresponding quantitative analysis of DC maturation after different treatments, n = 3. D) TNF-α and IL-6 levels in cell supernatants of different groups after various treatments, date are presented as mean ± SD, n = 3.

Cancer immunotherapy has been an important adjunct in combating MDR-positive tumors, thus, the amplified ICD synergistically triggered by chemotherapy and ER stress induction was then investigated. Translocation of CRT and HMGB1 is an essential signal in ICD. As depicted in **Figure 4A**, the cells had the highest CRT exposure on the cell membrane after Ag-TF@^P^DOX treatments. Moreover, Ag-TF@^P^DOX induced the most significant HMGB1 release from the nucleus to the extracellular space compared to that in the control and TF@^P^DOX groups, which demonstrated firmly that in combination with ER stress, chemotherapy can more easily trigger the release of DAMPs. ICD-associated immunogenicity activated by chemotherapy and ER stress was then evaluated by the Transwell assay, in which MCF-7/ADR cell debris obtained after different treatments and immature DCs were seeded in the upper and the lower Transwell chambers, respectively **(Figure 4B)**. After incubating the MDR cells with DCs, a slight increase in DC maturation, characterized by the upregulation of typical costimulatory molecules (CD11c^+^, CD80^+^, and CD86^+^), was observed in the TF@^P^DOX group compared with the control group. Notably, at the same dose of DOX, the increase in DC maturation in the Ag-TF@^P^DOX group was obviously higher than that in the TF@^P^DOX group **(Figure 4C)**. On the other hand, we detected the highest levels of secreted tumor necrosis factor alpha (TNF-α) and interleukin 6 (IL-6) in the Ag-TF@^P^DOX group, as shown in **Figure 4D**. The reason for this finding is probably explained by the fact that chemotherapy alone can only stimulate ICD through secondary or “collateral” ER stress effects, which were milder than the direct alteration of ER homeostasis induced by Ag NPs. Moreover, the combined action of ER stress and chemotherapy led to higher levels of ICD-associated immunogenicity, which is promising for future applications.

### Cellular Uptake and Tumor Spheroid Penetration

**Figure 5:**
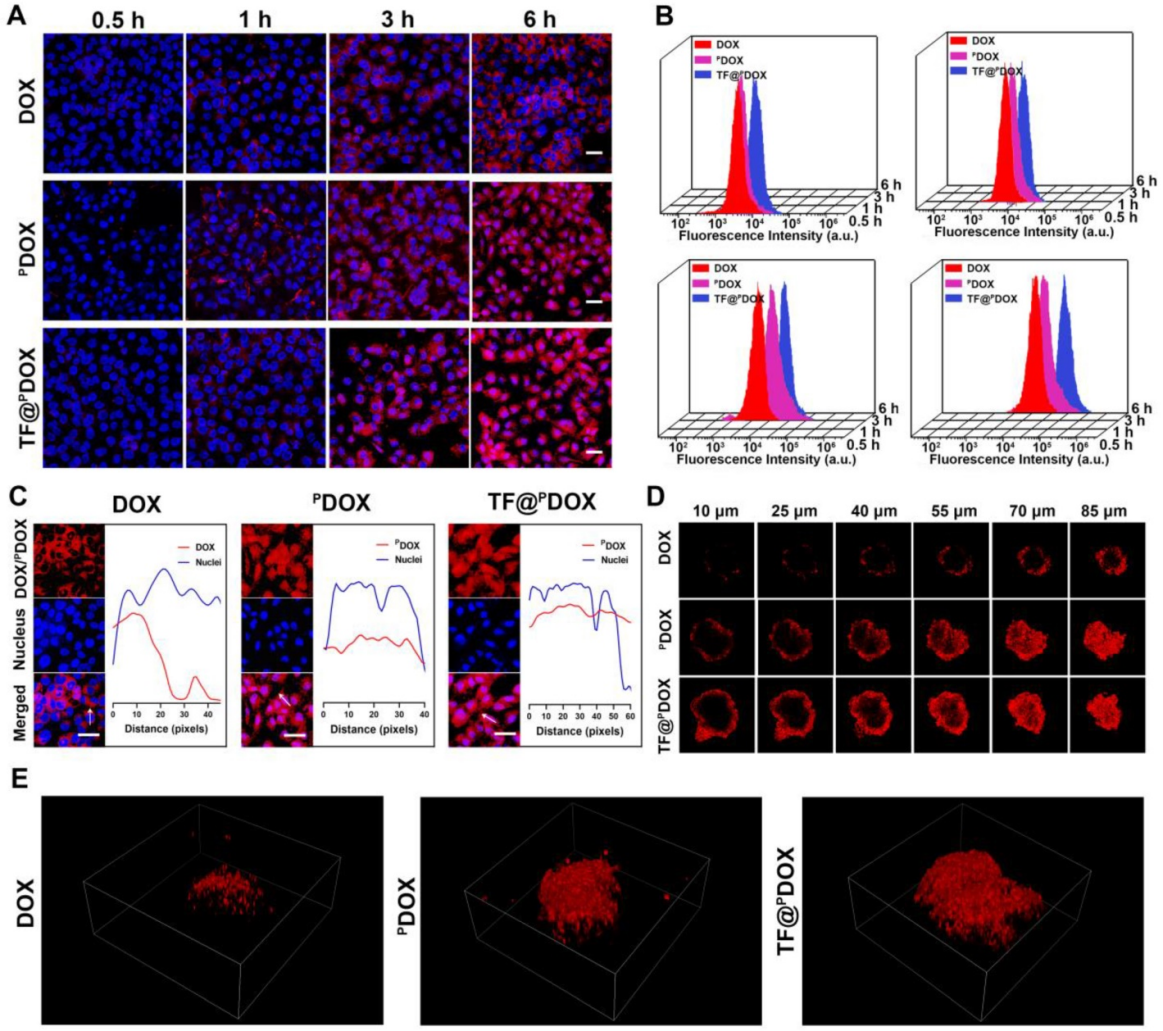
Intracellular uptake behavior of nanoagents. A) Confocal images of the MCF-7/ADR cells coincubated with DOX, ^P^DOX, and TF@^P^DOX for 0.5, 1, 3, and 6 h, respectively. DOX or ^P^DOX are represented by red fluorescence and nuclei are stained with DAPI. The scale bars are 50 μm. B) Flow cytometry analysis of MCF-7/ADR cells coincubated with DOX, ^P^DOX and TF@^P^DOX for 0.5, 1, 3, and 6 h. C) Nuclear localization observation and corresponding fluorescent profile analysis of DOX, ^P^DOX and TF@^P^DOX. The scale bars are 50 μm. D) Multiple level scans of MCF-7/ADR three-dimensional tumor spheroids for the penetration analysis of DOX, ^P^DOX, and TF@^P^DOX. E) Three-dimensional viewer based on three-dimensional reconstruction of the MCF-7/ADR spheroid models after coincubating with DOX, ^P^DOX, and TF@^P^DOX for 6 h.

An NLS sequence can guide substances to nuclei, which is thought to significantly amplify the effectiveness of chemotherapy in killing tumor cells; therefore, the cellular uptake behaviors of free DOX, free ^P^DOX, and TA-Fe^3+^-coated ^P^DOX in MCF-7/ADR cells were assessed via confocal laser scanning microscopy (CLSM). As shown in **Figure 5A,** increased amounts of free ^P^DOX and TF@^P^DOX emitting red fluorescence aggregated and entered the MCF-7/ADR cells after coincubation for 0.5, 1, 3, and 6 h; however, weaker fluorescence was observed around the cells in the free DOX group. The greater cell internalization efficiency probably resulted from the cell membrane-penetrating and nucleus-targeting abilities of the CPP. Subsequently, the nuclear delivery behavior was further investigated by observing the colocalization of DOX fluorescence with the nuclei. As depicted in **Figure 5C**, since free DOX can gradually diffused into cells after 6 h of coincubation, red fluorescence appeared mainly in the cytoplasm of MCF-7/ADR cells. Notably, both ^P^DOX and TF@^P^DOX colocalized extensively in not only the cytoplasms but also in the nuclei of MDR cells, clearly indicating that CPP-conjugated DOX can be successfully transported into nuclei driven by the NLS sequence. Furthermore, the quantitative results of cellular uptake were measured via flow cytometry analysis. With the prolonged incubation time, the ^P^DOX and TF@^P^DOX groups showed higher intracellular fluorescence intensity than the DOX group, which was consistent with the CLSM results. More interestingly, the fluorescence intensity of TF@^P^DOX was the strongest, even much higher than that of ^P^DOX **(Figure 5B)**. This result is probably explained by the fact that reduced P-gp expression may improve cellular drug accumulation, thereby enhancing fluorescence emission. In addition, the three-dimensional tumor spheroids formed by MCF-7/ADR cells imitated the *in vivo* status of MDR tumors, enabling the assessment of the permeability of the tested polypeptide. As shown in **Figure 5D,** the red fluorescence emitted from TF@^P^DOX and ^P^DOX was more clearly distributed in the tumor spheroids than that emitted from DOX after coincubation for 6 h. Additionally, we found that ^P^DOX enabled this versatile nanocomposite to move throughout the whole spheroid, while only a small amount of DOX adhered to the margin of the tumor spheroid, as determined via three-dimensional image reconstruction **(Figure 5E)**. Quantitative analysis revealed that the penetration depths of TF@^P^DOX and ^P^DOX were 33.23 μm and 31.31 μm, respectively, which were significantly greater than the depth of free DOX **(Figure S14)**. In contrast to general CPPs, which can easily penetrate the negatively charged cell membrane due to abundant cationic amino acids, the satisfactory penetrating behavior of CB5005 is probably attributed to its hydrophobic membrane-translocating characteristics that are derived from a membrane-permeable sequence (KLKLALALALA). This eventually facilitates the delivery of the nanosystem into the core of tumor tissue, possibly promoting the reversal of MDR.

### Cytotoxicity and Anticancer Effects *In vitro*

**Figure 6:**
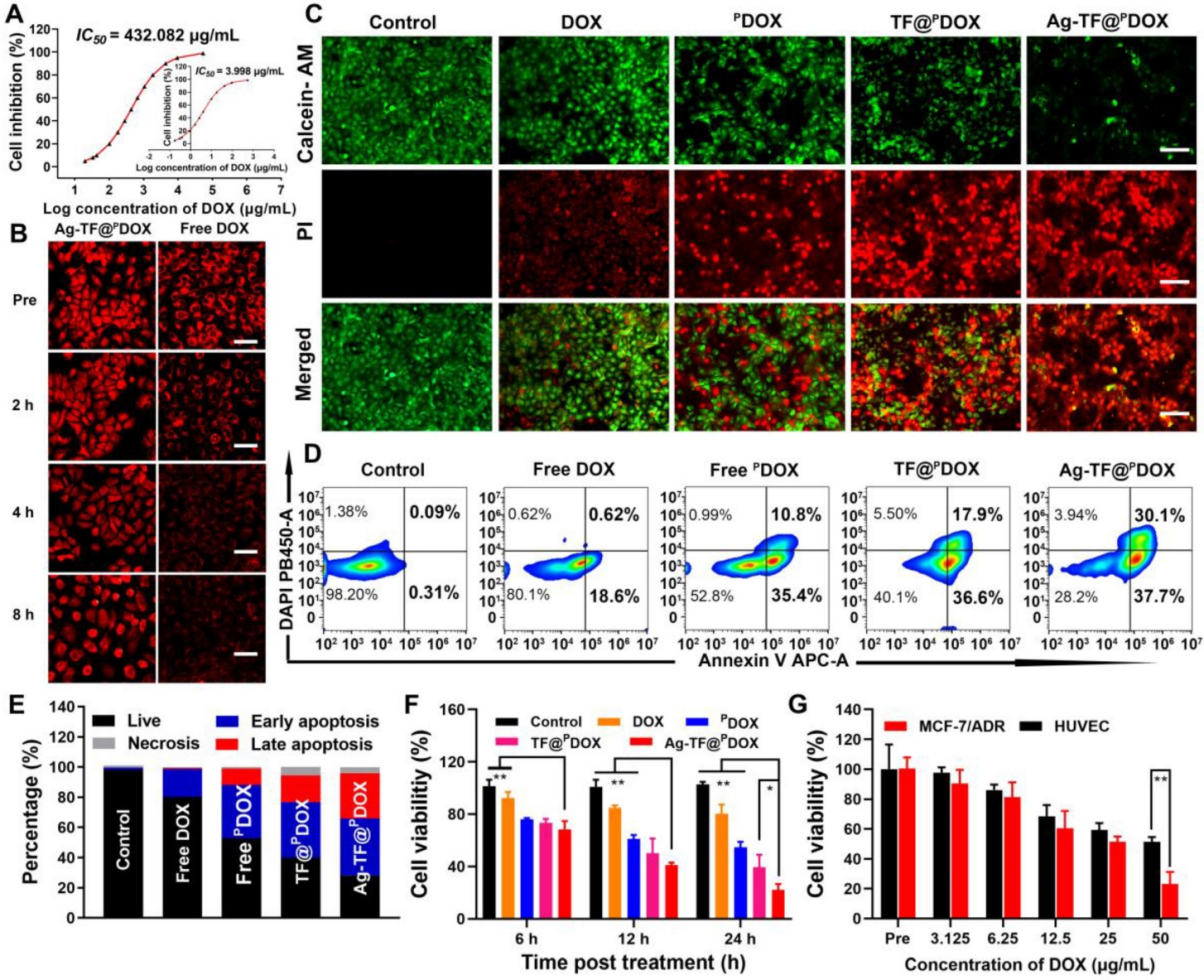
*In vitro* enhanced chemotherapeutic efficacy. A) IC_50_ curve of DOX against MCF-7/ADR drug resistant cell line (inset: IC_50_ curve against MCF-7 parental cell line). B) Intracellular DOX retention of MCF-7/ADR cells coincubated with Ag-TF@^P^DOX and free DOX after drug efflux, the scale bars are 50 μm. C) Fluorescent images of MCF-7/ADR cells costained with PI and calcein AM after corresponding treatments, death cells are represented by red fluorescence and live cells are represented by green fluorescence. The scale bars are 100 μm. D) Flow cytometry and E) corresponding quantitative analysis on the apoptosis levels of cells after corresponding treatments. F) Cell viabilities of MCF-7/ADR cells after incubating with corresponding formulations for 6, 12, 24 h, n = 3. G) Cell viabilities of MCF-7/ADR and HUVEC cells after incubating with Ag-TF@^P^DOX at different concentrations for 24 h, n = 3.

Before assessing the cytotoxicity of Ag-TF@^P^DOX *in vitro*, we initially detected the half-maximal inhibitory concentration (IC_50_) values of DOX against the MCF-7 parental cell line and drug-resistant cell line. The IC_50_ value was 108.07-fold higher in MCF-7/ADR cells than in MCF-7 cells, suggesting the successful construction of a DOX-resistant cell line **(Figure 6A)**. Drug efflux is one of the most important mechanisms of MDR. Fortunately, enhanced drug retention was expected, benefiting from P-gp downregulation and the nuclear targeting specificity of Ag-TF@^P^DOX. To verify this supposition, MCF-7/ADR cells were first incubated with free DOX and Ag-TF@^P^DOX for 4 h, and then, the medium with the DOX or Ag-TF@^P^DOX compounds was replaced with a drug-free solution to observe intracellular DOX retention. As shown in** Figure 6B** and **Figure S15**, the DOX level of MCF-7/ADR cells was notably decreased in the free DOX group after 8 h of removal of the drug, however, extensive DOX fluorescence was still visible in the cytoplasm and nucleus of the MCF-7/ADR cells treated with Ag-TF@^P^DOX. These findings indicated that the drug efflux rate was markedly inhibited in the cells incubated with the novel nanocomposite, which directly enhanced the anticancer effect. A live/dead cell dual-staining assay was then performed. Despite some fluorescence overlap between DOX/^P^DOX and propidium iodide (PI), the large number of live cells emitting green fluorescence in the DOX group indicated minimal damage caused by commonly used chemotherapeutic agents, which can be ascribed to the drug-resistant nature of these MDR cells. Cell viability was decreased to a certain degree after treatment with ^P^DOX and TF@^P^DOX. Notably, strong red fluorescence emitted by apoptotic cells was observed in the Ag-TF@^P^DOX group, demonstrating that this treatment leads to significantly greater cytotoxicity than the other treatments **(Figure 6C)**. These findings were also confirmed by flow cytometry **(Figures 6D** and** 6E)**. Similar to the results of live/dead dual-staining assays, a high apoptosis rate of up to 67.8% was detected in the Ag-TF@^P^DOX group. We next evaluated the anticancer efficacy of different formulations at various time intervals by the Cell Counting Kit-8 (CCK-8) assay. Notably, the cell viabilities in the TF@^P^DOX and Ag-TF@^P^DOX groups decreased drastically as the incubation time was prolonged; however, the similar trends in the free DOX and ^P^DOX groups were less drastic **(Figure 6F)**. We speculated that the unremarkable responses were due to the process by which the drugs were released from the nanosystems; free drugs that can diffuse directly into the cytoplasm induced a faster response than the nanosystems. In addition, the cytotoxicity induced by TA-Fe^3+^ and Ag-TF@^P^DOX was evaluated in human umbilical endothelial cells (HUVECs) and MCF-7/ADR cells. TA-Fe^3+^, with eco-friendly features, had only a negligible effect on the survival of both HUVECs and MCF-7/ADR cell lines, even at concentrations as high as 200 μg/mL, as shown in **Figure S16.** Remarkably, Ag-TF@^P^DOX induced significantly lower cytotoxicity in HUVECs than in MCF-7/ADR cells, possibly because the normal ATP levels in nontumor cells were unable to trigger sufficient decomposition of the nanosystems for complete drug release **(Figure 6G)**. These findings also suggested that the versatile nanocomposite has improved biocompatibility because of the TA-Fe^3+^ film coating.

### *In vitro* and *In vivo* PAI/MRI Profiles

**Figure 7:**
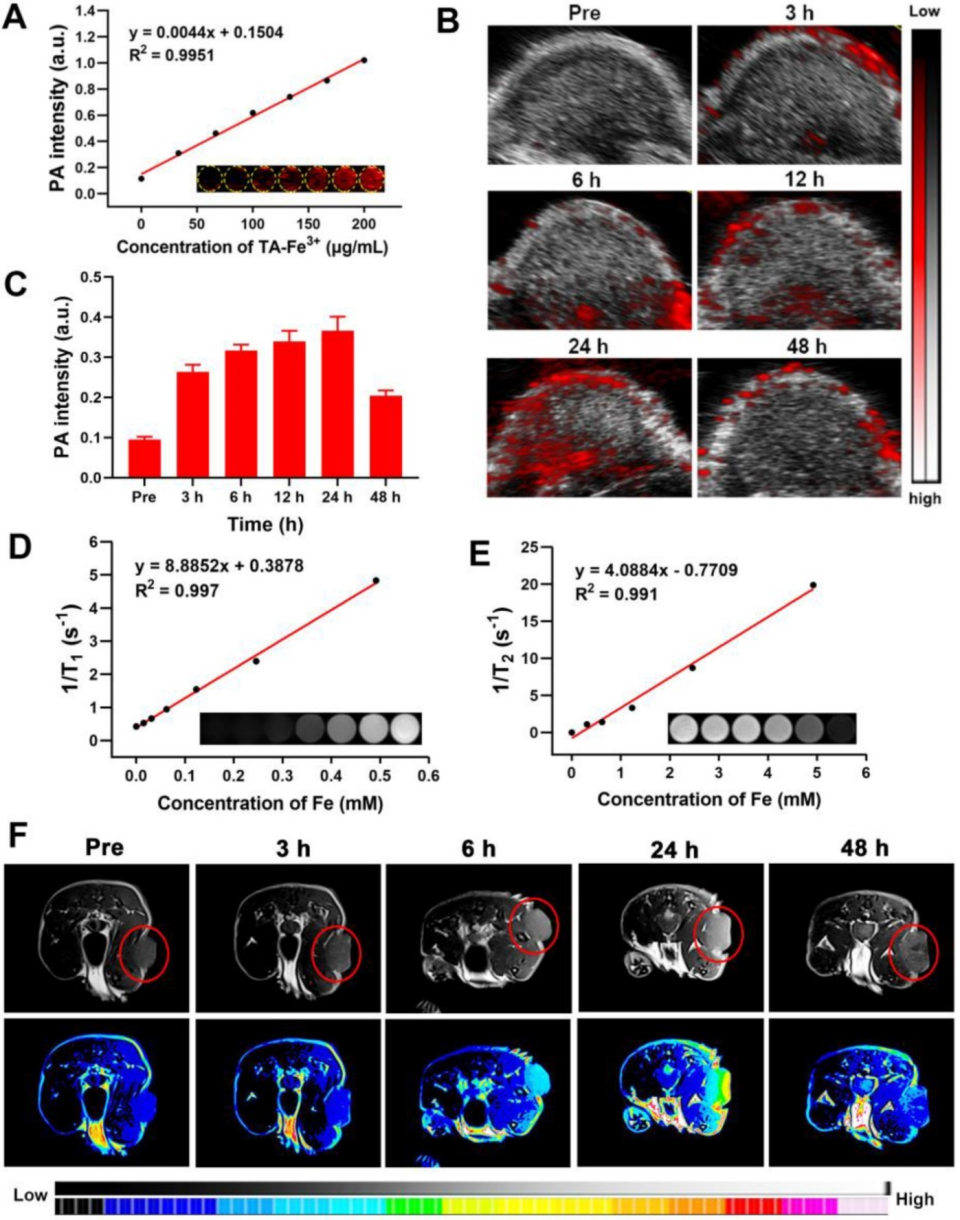
*In vitro* and *in vivo* PAI and MRI performance of Ag-TF@^P^DOX. A) *In vitro* PAI values and PAI images (inset) of Ag-TF@^P^DOX at different TA-Fe^3+^ concentrations. B) *In vivo* PAI images in tumor region after intravenous injection Ag-TF@^P^DOX at various time intervals and C) the corresponding photoacoustic signal values, n = 3. D) T_1_ and E) T_2_-weighted relaxation coefficient and *in vitro* corresponding MRI images (inset) for Ag-TF@^P^DOX. F) *In vivo* MRI images of MCF-7/ADR tumor-bearing mice after intravenous injection Ag-TF@^P^DOX at various time intervals.

Based on the good near-infrared absorption originating from the TA-Fe^3+^ network, Ag-TF@^P^DOX was expected to be a potential contrast-enhanced agent for use in PAI, a promising imaging modality because of its high sensitivity and noninvasiveness. After full-spectrum scanning in a photoacoustic system, an excitation wavelength of 690 nm was selected as the optimal value for the subsequent *in vitro* and *vivo* experiments **(Figure S17)**. Subsequently, Ag-TF@^P^DOX suspensions with different concentrations of TA-Fe^3+^ ranging from 0 to 200 μg/mL were scanned. The intensity of the photoacoustic signal was strengthened after the TA-Fe^3+^ concentration was increased, showing a good linear relationship (R^2^ = 0.995), as depicted in **Figure 7A**. To further confirm the PAI capability of the versatile nanocomposite *in vivo*, tumorous photoacoustic images of MCF-7/ADR tumor-bearing mice were obtained at predetermined time points, and the corresponding signal intensities were analyzed. The results showed that Ag-TF@^P^DOX clearly enhanced the photoacoustic signal at the tumor sites in a time-dependent manner, peaking at 24 h and decreasing over 48 h, which was mainly attributed to the clearance of the nanocomposite from the blood **(Figure 7B)**. Correspondingly, the quantitative analysis also revealed a similar increasing trend, suggesting that Ag-TF@^P^DOX with PAI capacity holds great promise for use in biomedical applications **(Figure 7C)**. As one of the most common imaging modalities for cancer diagnosis, MRI is widely used in the clinic due to its superior deep tissue penetrability and high spatial resolution. We initially observed the magnetic properties of the nanocomposite as determined by the magnetic hysteresis loop, which confirmed that Ag-TF@^P^DOX exhibited paramagnetic behavior, possibly originating from Fe^3+^ **(Figure S18)**. Considering this finding, we further systematically assessed the T_1_-weighted and T_2_-weighted MRI performances of the nanocomposites *in vitro* and *in vivo*. The longitudinal relaxation coefficient (r_1_) value of Ag-TF@^P^DOX, which corresponded to the slope of the fitted line in **Figure 7D**, was determined to be 8.8852 mM^-1^s^-1^ at iron concentrations ranging from 0 to 0.492 mM. Moreover, Ag-TF@^P^DOX at different concentrations also exhibited considerable negative enhancement in the T_2_-weighted MRI scans obtained *in vitro*, although the transverse relaxivity coefficient (r_2_) value was relatively low **(Figure 7E).** As the most commonly used MRI contrast agent, the Gd-based T1 contrast agent exhibited good longitudinal relaxation coefficient (r_1_) values ranging from 3.9 - 7.2 mM^-1^s^-1^ 42, 49**.** Considering the excellent longitudinal relaxivity coefficient of Ag-TF@^P^DOX, which can serve as an effective and safe MRI contrast agent, we chose T_1_-weighted imaging to evaluate the possibility of using the nanocomposite in MRI applications *in vivo*. Consistent with the *in vivo* results of PAI, a notably increasing T_1_-weighted enhancement was observed in the tumor region and decreased over 48 h, as demonstrated in** Figure 7F**, indicating efficient tumor accumulation of Ag-TF@^P^DOX through the EPR effect. The relative MRI signal intensity was substantially enhanced compared to that obtained with nontumor tissue and peaked at 24 h postinjection** (Figure S19)**. These findings demonstrated that well-designed nanocomposites accumulate in tumors with great efficiency and can be used in dual-modality imaging to achieve visual monitoring and theranostic guidance in one step.

### Therapy for DOX-resistant Tumors *In vivo*

**Figure 8:**
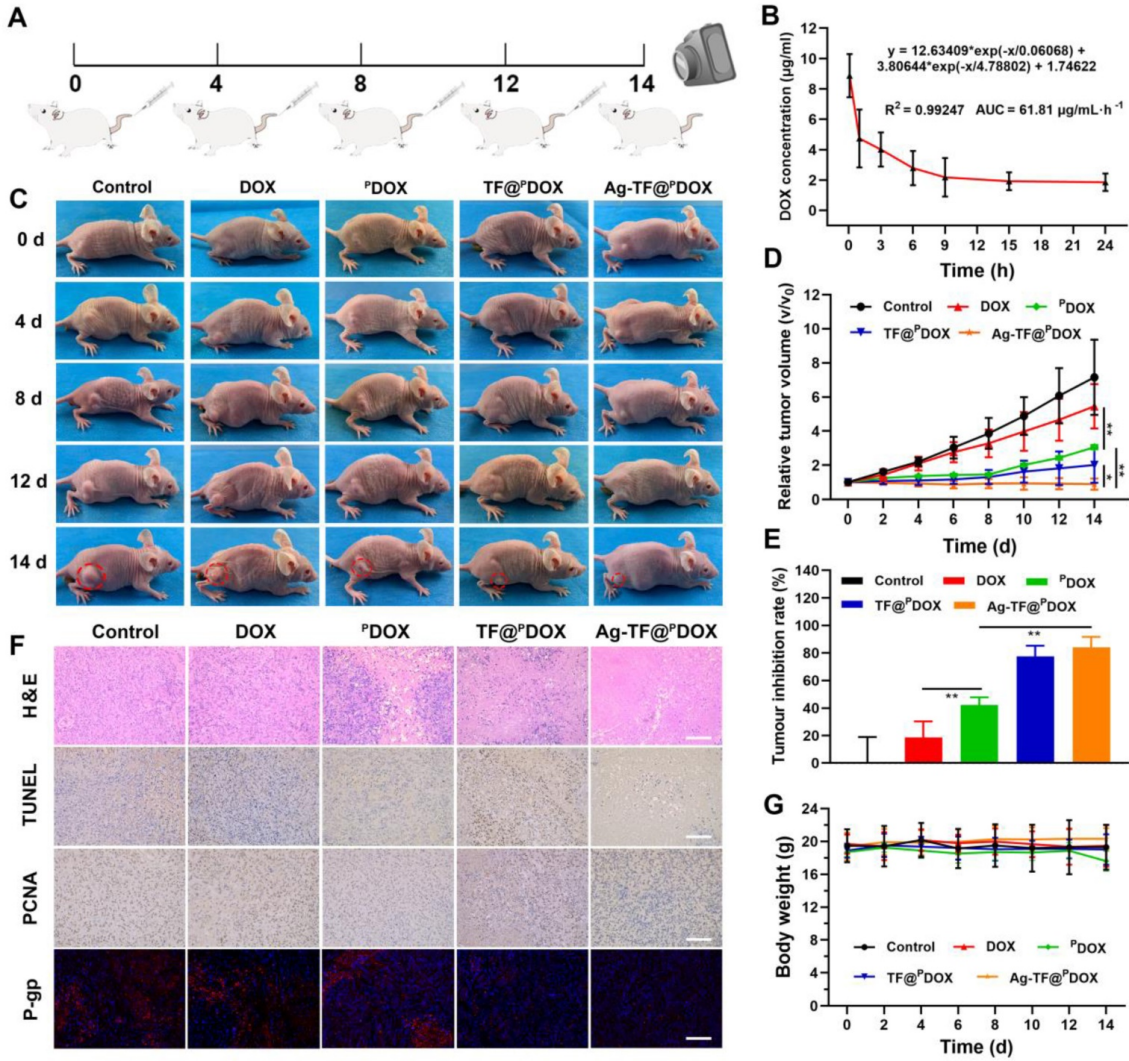
*In vivo* evaluations on enhanced chemotherapy-based anticancer efficacy. A) Schematic diagram of the administration time for *in vivo* therapy. All mice are intravenously administered with different agents on days 0, 4, 8, 12. B) Pharmacokinetics of Ag-TF@^P^DOX in blood circulation, n = 3. C) Digital pictures of MCF-7/ADR tumor-bearing mice of each group during 14-day observation after various treatments. D) Relative tumor growth curves of MCF-7/ADR tumor-bearing mice after different treatments, n = 5. E) Tumor inhibition rates of MCF-7/ADR tumor-bearing mice after administration of different agents, n = 5. F) H&E, TUNEL,PCNA, and P-gp staining of tumor sections from MCF-7/ADR tumor-bearing mice of various treatment groups, the scale bars are 100 μm. G) Body weight monitoring of mice in the different groups, n = 5.

**Figure 9:**
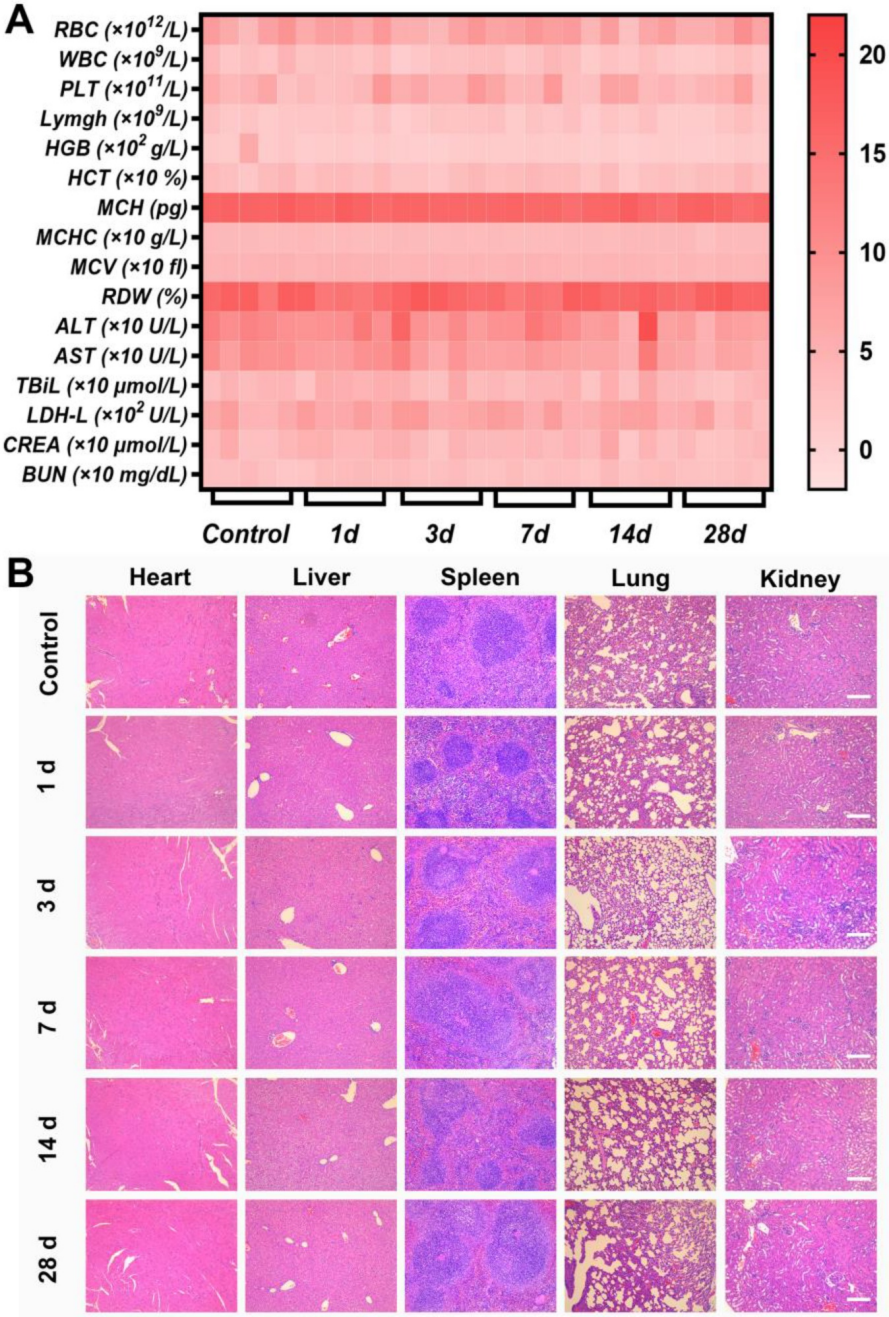
*In vivo* biosafety of Ag-TF@^P^DOX in the short and long term. A) Heat map of blood biochemistry and routine blood analysis in mice sacrificed on different days after intravenous administration of Ag-TF@^P^DOX, n = 5. B) H&E staining of the main organs in mice sacrificed at different time intervals after intravenous administration of Ag-TF@^P^DOX. The scale bars are 100 μm.

To investigate the performance of Ag-TF@^P^DOX in overcoming MDR *in vivo*, we monitored the changes in tumor volume for 14 days in MCF-7/ADR tumor-bearing mice, which were randomly allocated into five groups to receive different treatments **(Figure 8A).** The pharmacokinetics of DOX in the bloodstream were initially investigated after injection at a dose of 1.5 mg/kg. The area under the curve (AUC) was calculated to be 61.81 µg/mL·h^-1^, which was sufficient for drug effectiveness and biosafety** (Figure 8B)**. In addition, quantitative biodistribution of the nanocomposite in major organs (i.e., heart, liver, spleen, lung, and kidney) was evaluated **(Figure S20)**. The amounts of DOX in heart, spleen, and kidney were almost undetectable after 24 h postinjection. This suggests the remaining DOX in these organs have been metabolized, which guarantees desirable biosafety. In terms of antitumor efficacy *in vivo*, a limited inhibitory effect was detected in mice injected with DOX during 14 days of observation, which was similar to the results of *in vitro* testing. In comparison, the best antitumor efficacy was achieved in the group receiving Ag-TF@^P^DOX, which not only suppressed the rate of tumor growth but also reduced the tumor volume by the end of the treatment period, as shown in **Figures 8C and D**. Furthermore, at the end of observation, the tumor inhibition rate was calculated to be 84.0% greater in the Ag-TF@^P^DOX group than in the control group, confirming that chemotherapy enhanced by a nucleus-targeting strategy and ER stress can eradicate drug-resistant tumors **(Figure 8E)**. In addition, hematoxylin and eosin (H&E), TdT-mediated dUTP nick-end labeling (TUNEL), and proliferating cell nuclear antigen (PCNA) staining of tumor sections were performed. High apoptosis and necrosis rates were found in the tumors treated with Ag-TF@^P^DOX, while only slight damage was found in tumors treated with DOX or saline. The representative apoptosis-positive cells (dark brown) observed by TUNEL staining was similar to that observed with H&E staining. In contrast, PCNA staining of the tumors revealed an inverse trend, which reflected the cell proliferation rate. Remarkably, immunofluorescence staining of P-gp retained the same features *in vitro*, which showed the lowest P-gp expression in tumors treated with Ag-TF@^P^DOX **(Figure 8F)**. Histological examination of the major organs revealed no pathological damage or inflammatory lesions during the *in vivo* treatments **(Figure S21)**. Additionally, the body weights of the mice did not significant differ during the therapy period, as shown in **Figure 8G.** To evaluate the systemic toxicity of the nanocomposite *in vivo*, the systemic toxicity of Ag-TF@^P^DOX *in vivo* was also assessed in healthy Kunming mice through histopathological and hematological analyses. All the mice administered Ag-TF@^P^DOX showed negligible variations in both the short and long term compared to those in the control group **(Figure 9A)**. H&E staining of the major organs in Kunming mice showed neither detectable histomorphological damage nor an abnormal inflammatory response *in vivo* at any of the time points **(Figure 9B)**. Moreover, blood hemolysis of Ag-TF@^P^DOX was investigated as a safety evaluation. As shown in **Figure S22**, Ag-TF@^P^DOX encapsulating various concentrations of ^P^DOX induced little hemolysis (< 3%), revealing the acceptable biocompatibility of the nanocomposite.

### Anti-metastasis Efficacy of Combined Chemotherapy and Immunotherapy

**Figure 10:**
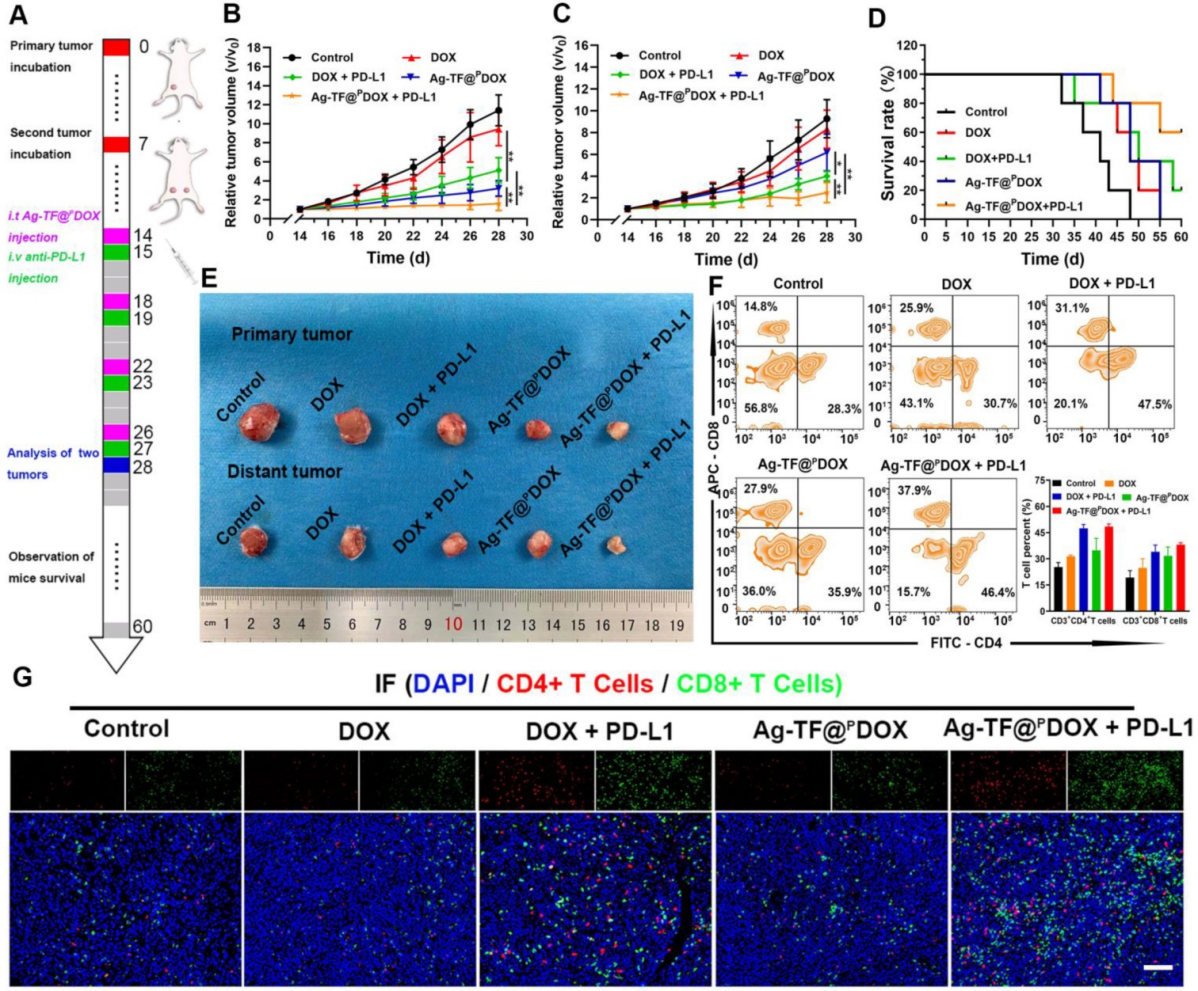
*In vivo* anticancer efficacy and immune activation initiated by nanocomposite-mediated ICD synergizing with PD-L1 blockade. A) Schematic on the administration time for *in vivo* therapy. Relative tumor growth curves of B) primary and C) distant tumors of 4T1 tumor-bearing mice after various treatments, n = 5. D) Survival curves of 4T1 tumor-bearing mice in each group during 60-day observation. E) Digital pictures of 4T1 tumors after receiving different treatments. F) Flow cytometry and corresponding quantitative analysis of T cells infiltration after different treatments, n = 3. G) Immunofluorescence images of CD4^+^ (red) and CD8^+^ (green) T cells in distant tumors after different treatments. The scale bars are 200 μm.

Tumor cells and the extracellular matrix constitute a complex immunosuppressive microenvironment, which dampens the response of solid tumors to immunotherapy. To amplify immune responses originating from the ICD effect, PD-1/PD-L1 checkpoint blockade, which can greatly attenuate the immunosuppressive status by preventing the interaction of PD-1 and PD-L1, was applied. Briefly, 4T1 tumor models that mimic bilateral tumors (primary and distant) were established in Balb/c mice, which were injected with the Ag-TF@^P^DOX nanocomposite and anti-PD-L1 antibody via the tail vein. During the therapeutic period, the tumor volume was recorded every other day as presented in **Figure 10A**. In the primary tumors, DOX treatment alone showed an unsatisfactory effect on tumor growth, while DOX + PD-L1 had a moderate inhibitory effect on tumor growth. Notably, both Ag-TF@^P^DOX and Ag-TF@^P^DOX + PD-L1 greatly inhibited tumor growth within 14 days of treatment **(Figure 10B)**. The tumor volumes of distant tumors were partially suppressed in the Ag-TF@^P^DOX group and DOX + PD-L1 group. Compared with the effects of DOX treatment alone, Ag-TF@^P^DOX + PD-L1 delayed distant tumor proliferation most obviously, as the tumor volume increased by only 2.49-fold compared to the original volume. Massive apoptosis and necrosis were found in the distant tumors of the Ag-TF@^P^DOX + PD-L1 group, as indicated by H&E staining, which was consistent with its effect on tumor growth** (Figure S23).** The greater efficacy of distant tumor suppression was probably derived from the synergistic effect of ICD and the PD-1/PD-L1 blockade (**Figures 10C, 10E and S24**). The survival curve demonstrated that 60% of the tumor-bearing mice in the Ag-TF@^P^DOX + PD-L1 group survived past the 60-day monitoring period, in contrast to the mice in the four other groups, indicating that immunochemotherapy effectively improved the survival rate in the long term **(Figure 10D)**. Having confirmed the tumor inhibitory effects in the primary and distant tumors of Balb/c mice, we further investigated the mechanism underlying affecting systemic immunity by analyzing the T-cell proportion and DC maturation. On day 28 of the experiment, tumor tissues from three tumor-bearing mice of each group were dissected to prepare a single-cell suspension. The level of tumor-infiltrating T cells in the distant tumors was determined by staining for CD3^+^, CD4^+^ and CD8^+^ T cells, which were subsequently identified by flow cytometry. The results showed that the average percentage of CD4^+^ T cells in the Ag-TF@^P^DOX + PD-L1 group was 48.46%, which was significantly higher than that in the control group (25.26%). In addition, the percentage of CD8^+^ T cells in the distant tumors of the mice after Ag-TF@^P^DOX + PD-L1 treatment was significantly increased, to 38.38%, which was nearly 1.99-fold higher than the increase in the control group **(Figure 10F)**. Interestingly, the CD8^+^ T-cell proportion in tumors treated with Ag-TF@^P^DOX alone was obviously increased. We speculated that mature DCs activated by ICD presented antigens to T cells and thus enhanced the adaptive immune responses, which were characterized by the formation of CTLs. Additionally, a similar trend was observed in tumor tissue slices as determined by the immunofluorescence assay **(Figure 10G).** Considering this fact, DC maturation in primary tumors was measured. The levels in the groups administered Ag-TF@^P^DOX and Ag-TF@^P^DOX + anti PD-L1 were significantly higher than those in the other groups **(Figure S25)**. This high DC maturation level demonstrated that Ag-TF@^P^DOX can efficiently kill cancer cells, whose debris releases tumor-associated antigens *in situ* that display an “autologous cancer vaccine-like” function. Moreover, the secretion of cytokines produced via helper T cells, such as IL-6, TNF-α, and interferon gamma (IFN-γ), which can enhance the activation of the innate immune response, was measured by ELISA. The results demonstrated that, consistent with the activation of tumor-infiltrating T cells, the highest secretion levels of the aforementioned cytokines were observed in the groups treated with Ag-TF@^P^DOX + PD-L1, implying successful initiation of the systemic immune response **(Figure S26)**.

## Conclusions

In summary, this study reports a theranostic nanocomposite aimed at reversing the MDR and metastasis of breast cancer by concurrently achieving ER stress and cell-nucleus penetration to enhance tumor immunochemotherapy. To create a nanosystem with potent anticancer activity, Ag NPs were introduced into the nanosystem, resulting in a two-pronged attack via uncontrolled ER stress, which not only triggered enhanced ICD stimulation but also sensitized MDR cells to chemotherapy by downregulating P-gp expression. Moreover, we conjugated a novel peptide with a cell membrane-penetrating and nuclear localization sequence to DOX, improving both cellular internalization and intranuclear accumulation. After surface TA-Fe^3+^ engineering, the nanocomposite featured ATP-responsive disassembly and ATP depletion properties to improve biocompatibility and decrease ATP-dependent drug efflux. Additionally, based on the good NIR absorption and paramagnetic behavior, Ag-TF@^P^DOX with imaging potential is promising as a dual-mode (PAI/MRI) contrast-enhanced agent for the implementation of visual monitoring and theranostic guidance in one step. This design of nanocomposites to induce ER stress and nuclear targeting, and sensitize cells to immunochemotherapy offers a novel strategy for combating MDR and metastasis at the subcellular level.

## Materials and Methods

**Materials.** Doxorubicin was purchased from Aladdin Inc. (Shanghai, China). TA and ferric chloride hexahydrate (FeCl_3_.6H_2_O) were obtained from Sigma-Aldrich Chemical Co. (St Louis, USA). Amino-modified silver nanoparticles were purchased from Xi'an Ruixi Biological Technology Co., Ltd (China). P-gp rabbit pAb was purchased from ABclonal (Wuhan,China). CB5005 (CKLKLALALALAVQRKRQKLMP) was synthesized by Chinapeptide Co., Ltd (Shanghai, China). PI, calcein-AM, and CCK-8 were obtained from Dojindo (Japan); MCF-7, MCF-7/ADR, HUVECs and TNF-α, IFN-γ, and IL-6 ELISA kits were purchased from Uscn Life Science (China), anti-CD3^+^-PE, anti-CD8a^+^-APC, anti-CD4^+^-FITC, anti-CD11c^+^-APC, anti-CD80^+^-FITC, and anti-CD86^+^-PE were purchased from Biolegend, (California, MA). Female BALB/c mice and BALB/c-nude mice were purchased from Weitonglihua Biotechnology Co., Ltd. (Beijing, China).

**Synthesis of CPP-conjugated DOX.** DOX (0.18 mmol) was first dissolved in 5 mL of dimethyl formamide, followed by the addition of 0.2 mmol 6-maleimidocaproic acid succinimidyl ester and 0.37 mmol triethylamine. After 1 h of reaction at room temperature, the reaction solution was concentrated by a vacuum-rotary evaporation procedure, and a large amount of frozen ethyl ether was poured into the precipitating liquid. The products were collected, and denoted as DOX-MAL. Then, 4.0 μmol of CB5005 was dissolved in 5 mL of dimethyl formamide, followed by the addition of 4.4 μmol DOX-MAL. The reaction solution was reacted at room temperature for 6 h and concentrated under vacuum-rotary evaporation. Frozen ethyl ether was poured into a reaction solution and then collected. The product was purified by fast liquid chromatography and vacuum-rotary evaporation to obtain the final CPP-conjugated DOX.

**Preparation of Ag-TF@^P^DOX.** Briefly, 4.4 mg of ^P^DOX (involving 800 μg DOX) was dissolved in 200 μL of DMSO solution. Then, 200 μL of the as-prepared DOX/DMSO solution was poured into 8 mL of deionized water under continuous sonication (65 W, 15 s). Next, 80 μL of TA (40 mg/mL) and 80 μL of FeCl_3_·6H_2_O (10 mg/mL) aqueous solutions were added sequentially to the above solution under constant sonication and vortexed followed by neutralization using 1 μM NaOH solution. Then, 40 μL of the Ag NP solution (0.1 mg/mL) was added to 1 mL of the obtained product. After stirring, the Ag-TF@^P^DOX was finally obtained after rinsing with deionized water by centrifugation (13,000 rpm, 25 min). A similar method was applied for the preparation of TF@^P^DOX except without the addition of sliver nanoparticle solution.

**ER Stress Detection.** To determine whether ER stress could be induced by Ag-TF@^P^DOX, 1 × 10^6^ MCF-7/ADR cells per well were first seeded for 24 h of incubation. Then, the culture medium containing TF@^P^DOX and Ag-TF@^P^DOX (DOX: 50 μg/mL) was incubated for another 4 h incubation. After that, the cells were lysed with RIPA buffer containing a protease inhibitor cocktail. The cell lysates were collected from the lysates at 10000 rpm for 10 min and the protein concentrations were determined using an enhanced BCA Protein Assay Kit. Equal amounts of protein were electrophoresed in a 10% sodium dodecyl sulfate-polyacrylamide gel and then transferred onto nitrocellulose membranes. The membranes were blocked with 5% nonfat dry milk for 2 h at room temperature and then incubated overnight at 4 ℃ with ER-related antibodies. The membranes were subsequently incubated with a horseradish peroxidase-conjugated secondary antibody (dilution 1:2000) for 1 h. Protein bands were visualized using the an enhanced chemiluminescence western blotting detection kit. Cytoplasmic Ca^2+^ levels were determined by staining with Fluo-8. Briefly, MCF-7/ADR cells were cultured at a density of 1 × 10^5^ in a laser confocal cell-culture dish for 24 h. Then, the cells were treated with TF@^P^DOX and Ag-TF@^P^DOX suspensions with equivalent DOX concentrations for 0, 1, 2, and 4 h. The cells were stained with Fluo-8 working fluid for 1 h at 37 ℃ and the cytoplasmic Ca^2+^ level was detected both by CLSM after washing twice.

**Cell Culture and Animal Model.** MCF-7/ADR cells were maintained in Roswell Park Memorial Institute (RPMI)-1640 medium containing 10% fetal bovine serum (FBS, Bioind, Israel), 1% penicillin/streptomycin (Beyotime Biotech, China), and 0.5 μg/ml DOX solution. MCF-7 cells and HUVECs were maintained in RPMI-1640 medium containing 10% FBS and 1% penicillin/streptomycin. All the cells were incubated at 37 ℃ in a humidified atmosphere containing 5% CO_2_. All experiments involving mice were performed in accordance with the ethical standards of Chongqing Medical University. To establish MDR tumors, MCF-7/ADR cells (1 × 10^7^ cells per mouse) suspended in a sterile PBS solution (100 μL) were inoculated into the right flank of each nude mouse. To establish an orthotopic murine breast cancer model with spontaneous metastasis, 4T1 cells (1.5 × 10^6^ cells per mouse) suspended in a sterile PBS solution (100 μL) were subcutaneously inoculated under the right breast pad of each Balb/c female mouse as the primary tumor. Seven days later, 4T1 cells were inoculated under the left breast pad as distant tumors. The volume of tumors was calculated according to the following formula as 0.5 × length × (width)^2^.

**Anticancer Effect *In vitro*.** To observe the intracellular retention of Ag-TF@^P^DOX and DOX, MCF-7/ADR cells at a density of 1 × 10^5^ were initially maintained in a laser confocal dish for 24 h of incubation. Then the culture medium was replaced with the medium containing Ag-TF@^P^DOX and DOX and incubated for another 4 h. After that, the cells were washed three times with PBS and treated with fresh RPMI-1640 culture medium for another 2, 4, 6, and 8 h of incubation, respectively. At the predetermined time point, the cells were rinsed to remove the effluent DOX and then observed by CLSM to detect drug retention. To determine the cytotoxicity *in vitro*, the MCF-7/ADR cells were divided into the following 5 groups: (i) control, (ii) DOX, (iii) ^P^DOX, (iv) TF@^P^DOX, and (v) Ag-TF@^P^DOX. First, MCF-7/ADR cells at a density of 1 × 10^5^ cells per well were maintained in a confocal dish. Twenty-four hours later, fresh culture medium containing agents with an equivalent concentration of DOX (50 μg/ml) was added and coincubated with the cells for another 24 h. The therapeutic effects were detected by inverted fluorescence microscopy (Olympus IX53, Canada) after costaining with a dye solution of calcein AM (2 μM) and PI (10 μM) for 10 min at 37 °C. The cells above were also analyzed by cellular apoptosis assay. The treated cells were collected in 200 μL of PBS buffer and then stained with DAPI-PB450 and Annexin V-APC for 15 min. The stained cells were analyzed using flow cytometry. In addition, the cell viabilities were evaluated by CCK-8 assay. Initially, the MCF-7/ADR cells at a density of 1 × 10^4^ were seeded in 96-well culture plates for 24 h. Then, the culture medium was replaced with the different media in the following 5 groups: (i) control, (ii) DOX, (iii) ^P^DOX, (iv) TF@^P^DOX, and (v) Ag-TF@^P^DOX with an equivalent concentration of DOX (50 μg/ml). After a predetermined time of treatment (6, 12, and 24 h postinjection), the CCK-8 assay was used to evaluate the viability of cells in each group. To investigate the cytotoxicitiy of TA-Fe^3+^ and Ag-TF@^P^DOX against different cell lines, the HUVECs and MCF-7/ADR cells were initially seeded in 96-well culture plates for 24 h. Then, the culture medium containing TA-Fe^3+^ or Ag-TF@^P^DOX at different concentrations was added. After 24 h of treatment, the CCK-8 assay was used to evaluate the cell viability.

**MDR Tumor Growth Inhibition *In vivo***. To evaluate the *in vivo* MDR reversal efficacy of Ag-TF@^P^DOX, twenty-five MCF-7/ADR tumor-bearing nude mice were randomly divided into 5 groups (n = 5): (i) control, (ii) DOX, (iii) ^P^DOX, (iv) TF@^P^DOX, and (v) Ag-TF@^P^DOX. The corresponding formulations were intravenously administered to MCF-7/ADR tumor-bearing mice at an equivalent dose of 1.5 mg/kg DOX per mouse 4 times every 4 days. Saline solution (200 μL) was administered to mice in the control group. The tumor volume and weight changes of each mouse were monitored with a digital camera and recorded every other day during a 2-week observation window. The tumor volume changes were normalized using the relative tumor volumes (initial tumor volume (V_0_) / current tumor volume (V)). One mouse of each group was sacrificed on day 14, and the main organs and the targeted tumor tissues were excised and fixed for H&E staining. Tumor tissue was further stained with TUNEL, PCNA and P-gp.

**Statistical Analysis.** All data are expressed as the means ± standard deviation (SD) and were analyzed with SPSS 22.0 software. The statistical significance of difference between groups was compared using Student's t test and one-way ANOVA tests. Significance levels were demonstrated as *p < 0.05, **p < 0.01.

## Supplementary Material

Supplementary methods, figures and table.
