# Supplementary material for: Combating multidrug resistance and metastasis of breast cancer by endoplasmic reticulum stress and cell-nucleus penetration enhanced immunochemotherapy
Source: Theranostics. 2022 Mar 21;12(6):2987–3006. doi: 10.7150/thno.71693 (PMC8965496; doi:10.7150/thno.71693)
Supplement: Supplementary file 1 — Supplementary methods, figures and table. [file thnov12p2987s1.pdf]

Combating multidrug resistance and metastasis of breast cancer by endoplasmic reticulum stress and cell-nucleus penetration enhanced immunochemotherapy

Weixi Jiang[a,1], Li Chen[b,1], Xun Guo[a], Chen Cheng[a], Yuanli Luo[a], Jingxue Wang[a], Junrui Wang[c], Yang Liu[d], Yang Cao[a], Pan Li[a], Zhigang Wang[a], Haitao Ran[a], Zhiyi Zhou[e] and Jianli Ren[a]*

[a] Department of Ultrasound and Chongqing Key Laboratory of Ultrasound Molecular Imaging, the Second Affiliated Hospital of Chongqing Medical University, Chongqing 400010, P. R. China

[b] Department of Intensive Care Unit, the Second Affiliated Hospital of Chongqing Medical University, Chongqing 400010, P. R. China

[c] Department of Radiology, the Second Affiliated Hospital of Chongqing Medical University, Chongqing 400010, P. R. China

[d] Department of Gynaecology and Obstetrics, the Second Affiliated Hospital of Chongqing Medical University, Chongqing 400010, P. R. China

[e] Department of General practice of Chongqing General Hospital, University of Chinese Academy of Sciences, Chongqing 401147, P. R. China

* Corresponding author.

E-mail address: renjianli@cqmu.edu.cn (Jianli Ren)

[1] These authors contributed equally to this work.

Experimental Sections:

Characterization. The morphology of Ag-TF@^{P}DOX was observed using TEM (Hitachi H-7600, Japan) and SEM (Hitachi S-3400N,Japan) instruments. DLS (Malvern, NanoZS, UK) was used to measure the zeta potentials and size distribution. The absorption spectrum of Ag-TF@^{P}DOX, TF@^{P}DOX, ^{P}DOX, TA-Fe^{3+}, and Ag NPs were determined using UV spectroscopy (Shimadzu, UV-3600,Japan) to confirm successful loading of corresponding agents. UV spectroscopy was used to observe the absorption spectrum of ^{P}DOX and DOX at different concentrations. The synthesis of CB5005 was validated by MS (BAIIENS, AB5800, China). The synthesis of ^{P}DOX was validated by NMR (AVANCE III, Bruker,

Germany) and HPLC (Waters, ZQ2000,China). The standard curve of free ^{P}DOX was depicted by UV spectroscopy and HPLC to measure the amount of ^{P}DOX encapsulated into nanocomposite. The entrapment efficiency and content of ^{P}DOX were calculated by Equations (1) and (2).

$$(1)\ {}^{P}\text{DOX entrapment efficiency (\%)}=\frac{\text{Mass of total }{}^{P}\text{DOX - Mass of unentrapped }{}^{P}\text{DOX}}{\text{Mass of total }{}^{P}\text{DOX}}\times 100\%$$

$$(2)\ {}^{P}\text{DOX entrapment content (\%)}=\frac{\text{Mass of total }{}^{P}\text{DOX - Mass of unentrapped }{}^{P}\text{DOX}}{\text{Mass of total nanocomposite}}\times 100\%$$

Nucleus Targeting and Tumor Spheroid Penetrating Efficiency. Briefly, MCF-7/ADR cells at a density of 1×10^5 per well were maintained in a confocal culture dish. After 24 h incubation, the culture medium containing DOX, ^{P}DOX and, TF@^{P}DOX (DOX: 20 μg/ml) were added and coincubated for 0.5, 1, 3, and 6 h, respectively. To observe cellular uptake behavior, the cells were fixed in 4% formaldehyde for 15 min and washed with PBS. The cell nuclei were then stained with DAPI for 10 min. Finally, CLSM was performed to observe the fluorescent images and nucleus-targeting performance was

analyzed by Image J. In addition, the quantitative intracellular fluorescence of DOX, ^{P}DOX and TF@^{P}DOX for MCF-7/ADR cells at different time intervals were detected by flow cytometry (Becton Dickinson, USA). To establish three-dimensional spheroids of MCF-7/ADR cells, 1.5 $\times 10^5$ MCF-7/ADR cells were maintained in 6-well ultra-low adherent plates for 7 days formation. Next, the spheroids were coincubated with DOX, ^{P}DOX and TF@^{P}DOX for 6 h and observed by CLSM. Scans in multiple levels for three-dimensional tumor spheroids were performed to measure penetration depth.

P-gp Expression. To investigate drug resistance mechanism mediated by ER stress, P-gp expression was detected by flow cytometry. MCF-7/ADR cells were cultured at a density of 2 $\times 10^5$ cells in 6-well plates for 24 h. The cell culture medium was replaced with the medium containing with different concentrations of Ag NP (0, 0.125, 0.25, 0.5, 1 μg/ml) for another 4 h incubation. After that, the cells were washed twice and collected. Then, the cell suspension was stained with labeled anti-CD243 (MDR-1) antibody and analyzed by flow cytometry. To investigate the degree of P-gp inhibition by different agents, MCF-7/ADR cells were treated with RPMI-1640 culture medium, DOX, ^{P}DOX, TF@^{P}DOX, and Ag-TF@^{P}DOX

(DOX: 50 μg/ml) for 4 h. After coincubation, the cells were collected and P-gp expression was then analyzed by flow cytometry and western blotting.

Pharmacokinetics of Ag-TF@^{P}DOX. For the pharmacokinetics study, 20 μl blood of healthy Kunming mice (n = 3) were collected at predetermined time points (0.08, 1, 3, 6, 9, 15, 24 h postinjection) after intravenously injection of Ag-TF@^{P}DOX. Then, a 20 μl of serum sample was mixed with 100 ul of extraction solution (methanol: methylene chloride =1:1) followed by grinding for 30 min and centrifuging for 10 min at 15,000 g. After that, a 100 ul of supernatant was extracted and dried with nitrogen at 40 °C, which was then redissolved with 100 μl 70% acetonitrile. Finally, the samples were analyzed by HPLC.

Drug Release. 15 ml of Ag-TF@^{P}DOX suspension (DOX: 200 μg/ml), which was equivalently moved into three dialysis bags (MW cutoff 3,500 Da), were placed in centrifugal tubes containing 25 ml buffer solution involving different ATP content (0, 2, and 5 mM) with a 150 rpm stirring rate at 37 °C. At every predetermined time interval (pre, 0.5, 1, 3, 5, 8, 12,

18, 24, 36, and 48 h), 1 ml of buffer solution in the centrifugal tube was collected and replaced by 1 ml of fresh buffer solution. Finally, digital photographs of buffer solution were obtained and cumulative release ratios in three groups were calculated based on the corresponding absorbance of the UV spectrum.

ATP Depletion. Initially, we investigated whether TA-Fe^{3+} and TF@^{P}DOX could decrease ATP level in solution by an ATP assay kit. The solutions were prepared by mixing ATP solutions (1 mM) with deionized water, TA-Fe^{3+} at different concentrations (12.5, 25, 50, 100, 200 μg/ml), or TF@^{P}DOX (containing TA-Fe^{3+} at the same concentrations), respectively. After stirring for 6 h, the supernatant was extracted from respective solutions for ATP assay kit according to the manufacturer's protocol. To detect intracellular ATP content, MCF-7/ADR cells were maintained in 6-well plates at a density of 2×10^{5} per well. After 24 h incubation, the cells were treated with 1640 medium, 100 μg/ml TA-Fe^{3+} and TF@^{P}DOX (containing TA-Fe^{3+} at same concentration) for different time intervals. Finally, the cells were digested and collected at 1000 rpm for 5 min after counting and normalizing the numbers of cells in each group and disrupted by cell lysis buffer. The supernatant was used for ATP determination.

In Vitro and In Vivo Performance of PAI and MRI. To test the potential of Ag-TF@^{P}DOX as a PAI contrast agent in vitro, a 3% agarose gel phantom was initially constructed. Ag-TF@^{P}DOX suspensions involving different concentrations of TA-Fe^{3+} were measured using a LAZR photoacoustic imaging system (Vevo LAZR, Canada.) to obtain photoacoustic images and corresponding intensities. For in vivo PAI, MCF-7/ADR tumor-bearing mice (n = 3) were anesthetized with pentobarbital sodium followed by intravenous injection of the Ag-TF@^{P}DOX solution. Then, the photoacoustic images were captured at every predetermined time point (preinjection and, 1, 3, 6, 12，24 and 48 h postinjection) and the photoacoustic intensities in the tumor regions were recorded. The MRI performance of Ag-TF@^{P}DOX was detected by a 3.0 T MRI scanner equipment (Siemens, German). Ag-TF@^{P}DOX involving different concentrations of Fe were firstly prepared in Eppendorf tubes. The corresponding images were obtained by an MRI system with a gradient echo sequence and the T_1 and T_2 relaxation time within the regions of interest were measured. MCF-7/ADR tumor-bearing mice (n = 3) were established to evaluate MRI performance in vivo. The MRI images and signal intensities in the site of the tumor area

were captured at predetermined time point (preinjection and, 3, 6, 24 and 48 h postinjection) after intravenous injection of Ag-TF@^{P}DOX.

Anticancer Efficacy of Combined Chemotherapy and Immunotherapy. To observe DC maturation in vitro, a Transwell assay was used to coincubate MCF-7/ADR cells and DCs. Firstly, MCF-7/ADR cells were seeded in upper layer plate of the Transwell system until adherent and then replaced by RPMI-1640 culture medium, TF@^{P}DOX, and Ag-TF@^{P}DOX (DOX: 50 μg/ml) for 24 h, respectively. The supernatant was then extracted and centrifuged to collect cell debris, which were then resuspended in fresh medium. Subsequently, the residues of MCF-7/ADR cells obtained after different treatments were added into the lower layer plate for co-culturing with DCs for another 24 h. Finally, the DCs were stained with anti-CD11c^{+}-APC, anti-CD80^{+}-FITC, and anti-CD86^{+}-PE, and then analyzed by flow cytometry. To investigate immune responses in vivo, the 4T1 tumor-bearing mice were then randomly divided into 5 groups (n = 5): (i) control, (ii) DOX, (iii) DOX + PD-L1, (iv) Ag-TF@^{P}DOX, and (v) Ag-TF@^{P}DOX + PD-L1. The day was defined as day 0 when primary tumors were inoculated. From day 14, corresponding formulations were intratumorally injected into primary tumors of 4T1

tumor-bearing mice at the equivalent dose of 0.75 mg/kg DOX per mouse 4 times every 4 d. Saline solution (200 μl) was administrated with mice in the control group. Furthermore, anti-PD-L1 (1.5 mg/kg per mouse) were intravenously injected into the mice in DOX + PD-L1 and Ag-TF@^{P}DOX + PD-L1 groups on days 15, 19, 23, and 17. On day 28, three mice of each group were sacrificed for tumor dissection to measure the levels of DC maturation and T cells infiltration. Briefly, the tumors were harvested and treated with 0.002% DNase, 0.2% collagenase D, and 0.01% hyaluronidase to produce a single-cell suspension. The cell suspension of distant tumors was used to detect the proportions of tumor-infiltrating T cells ($CD3^+$, $CD4^+$, and $CD8a^+$ T cells) by flow cytometry and immunofluorescencel, and cell suspensions of primary tumors were used to determine DC activation ($CD11c^+$, $CD80^+$, and $CD86^+$ T cells). The levels of TNF-α, IFN-γ, and IL-6 were determined by using ELISA kit. In addition, the tumor volume changes and the survival curve of each group were recorded.

Biosatey Assay. To investigate the biosafety of Ag-TF@^{P}DOX in vivo, Ag-TF@^{P}DOX at a dose of 1.5 mg/kg DOX or saline solution was intravenously injected into thirty healthy Kunming mice that were sacrificed at predetermined time points (day 1, 3, 7, 14, and 28). Five mice that were administrated with the saline solution were set as the control group. The

blood samples were then collected for biochemistry and routine blood examination, involving L-lactate dehydrogenase (LDH-L), aspartate transaminase (AST), creatine kinase, alanine aminotransferase (ALT), urea nitrogen (BUN), creatinine (CREA), and total bilirubin. The staining of major organs with H&E was analyzed histologically.

Supplementary Figures

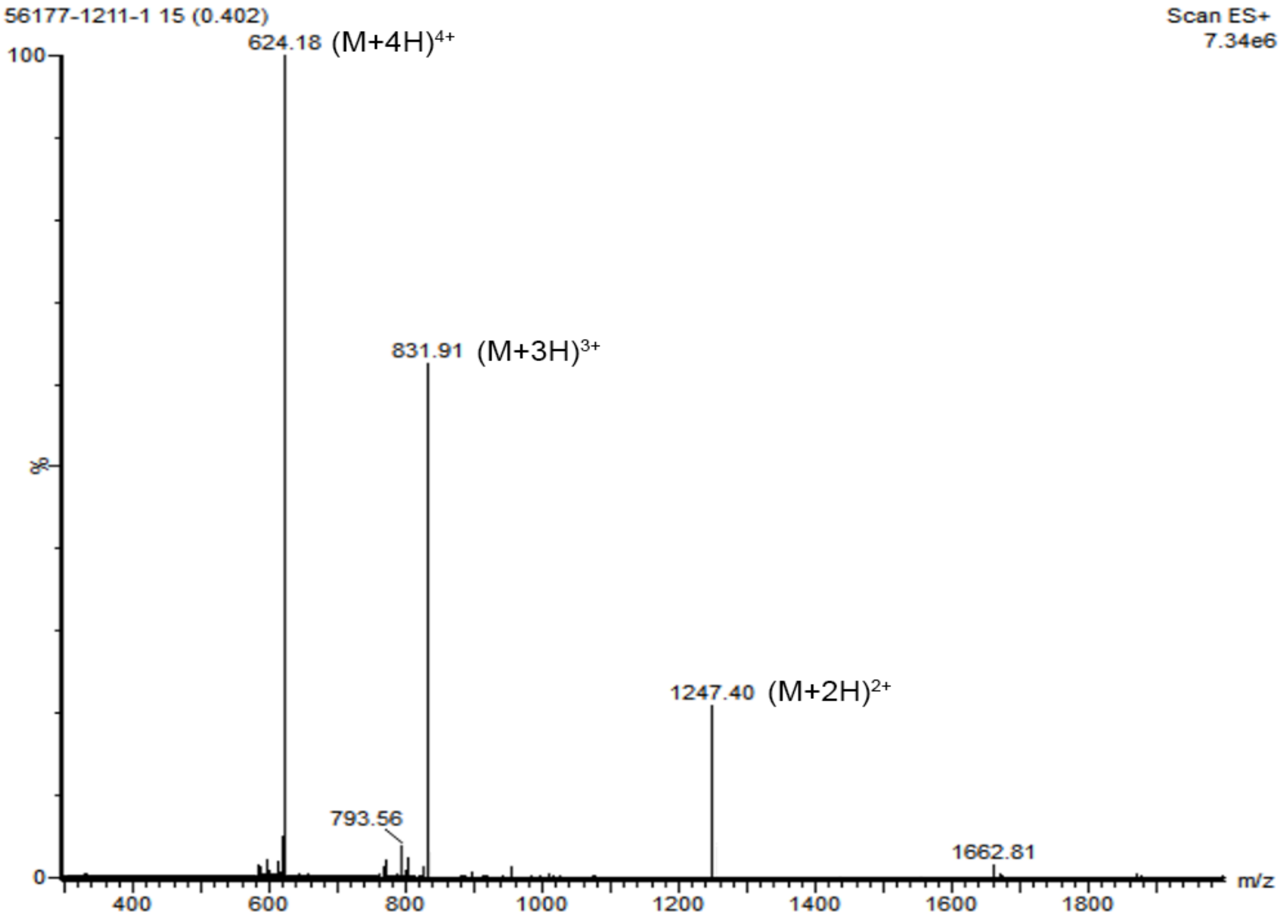

Figure S1. Mass spectrum of CB5005 (CKLKLALALALAVQRKRQKLMP).

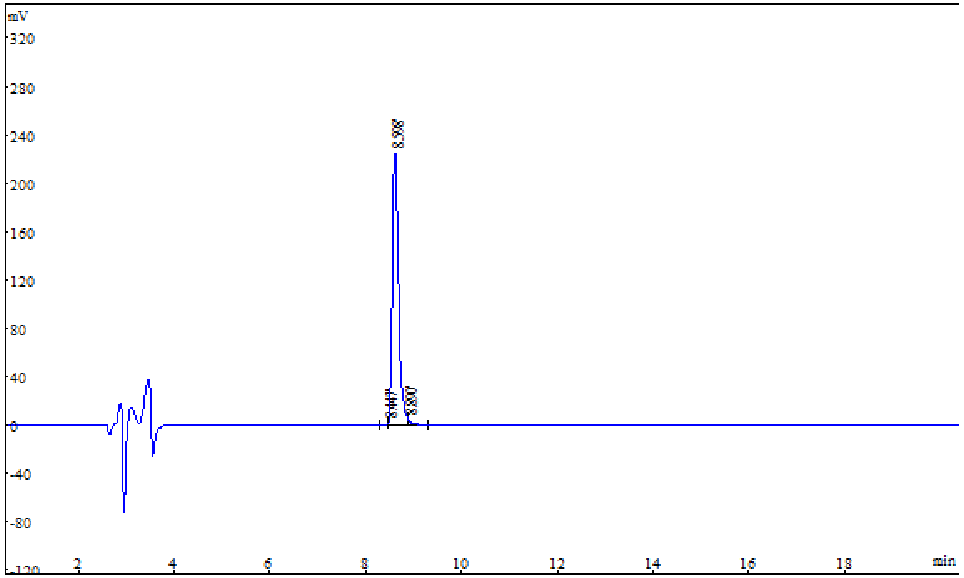

Rank	Time	Name	Conc.	Area
1	8.447		0.132	2543
2	8.598		98.04	1887860
3	8.890		1.832	35281
Total			100	1925684

Figure S2. HPLC analysis of CB5005.

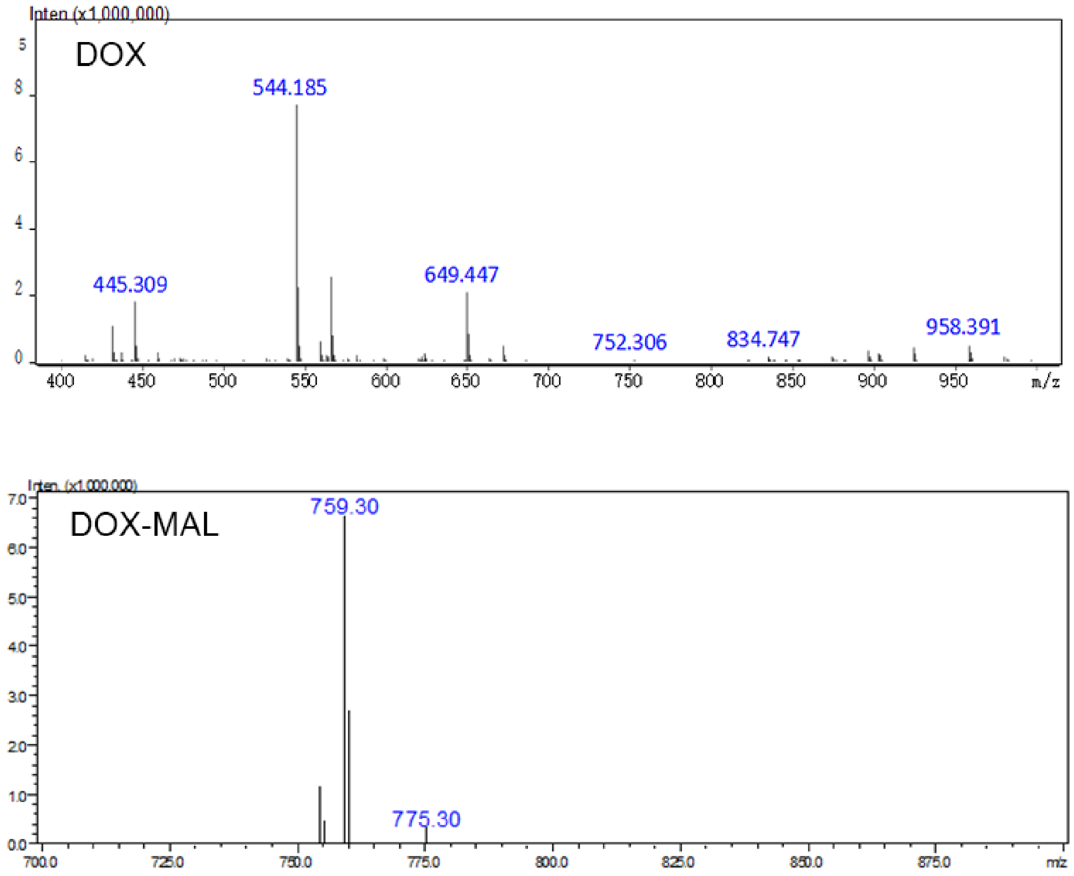

Figure S3. Mass spectrum of DOX and DOX-MAL.

a.

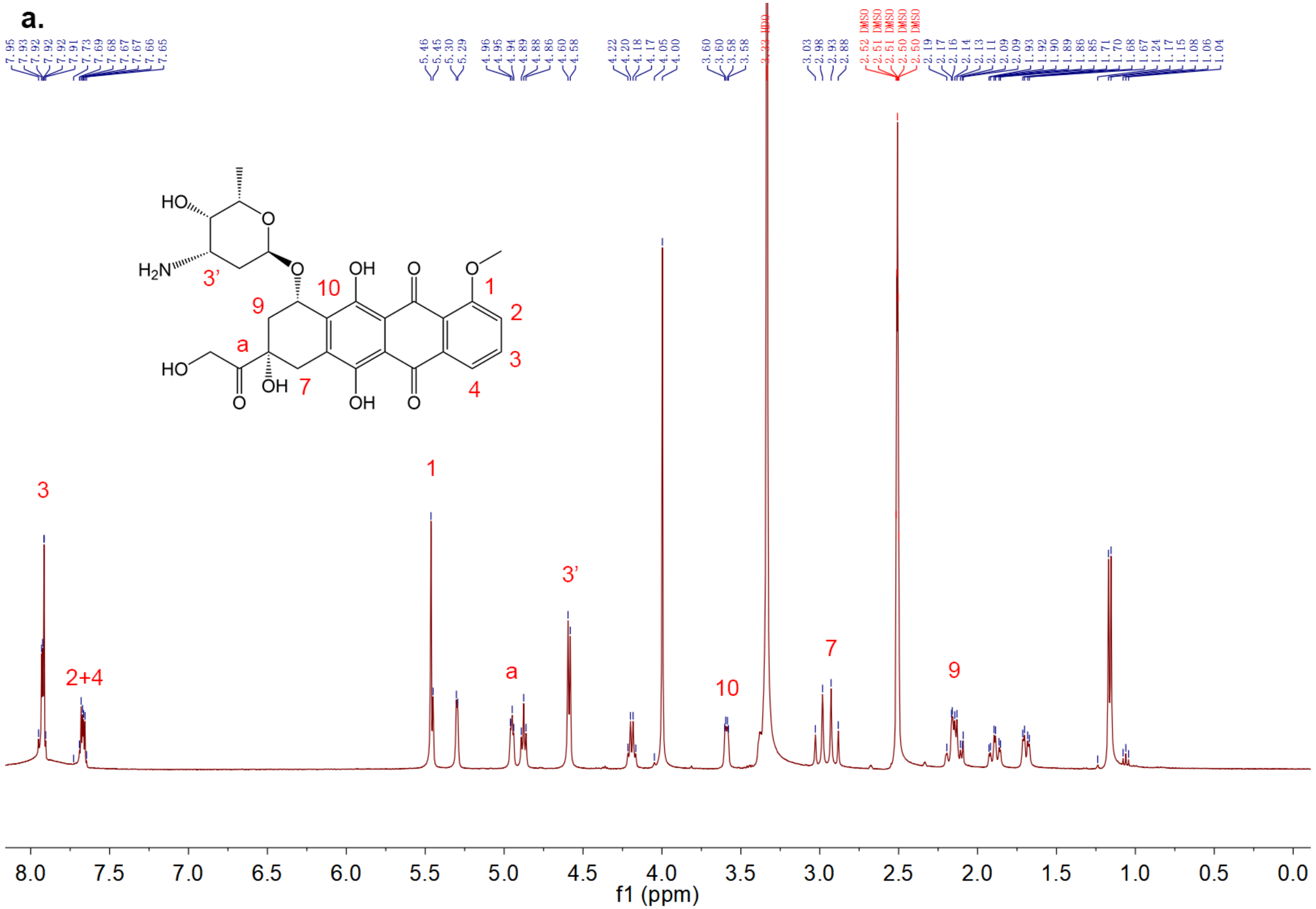
7.95 7.93 7.92 7.92 7.92 7.91 7.73 7.69 7.68 7.67 7.67 7.66 7.65
5.46 5.45 5.30 5.29
4.96 4.95 4.94 4.89 4.88 4.86 4.60 4.58
4.22 4.20 4.18 4.17 4.05 4.00
3.60 3.60 3.58 3.58
3.33 HDO
3.03 2.98 2.93 2.88
2.52 DMSO 2.51 DMSO 2.51 DMSO 2.50 DMSO 2.50 DMSO
2.19 2.17 2.16 2.14 2.13 2.11 2.09 2.09 1.93 1.92 1.90 1.89 1.86 1.85 1.71 1.70 1.68 1.67 1.24 1.17 1.15 1.08 1.06 1.04
HO
O
H2N
3'
OH
O
O
1
2
3
4
10
9
a
HO
O
OH
7
OH
O
3
2+4
1
a
3'
10
7
9
8.0 7.5 7.0 6.5 6.0 5.5 5.0 4.5 4.0 3.5 3.0 2.5 2.0 1.5 1.0 0.5 0.0
f1 (ppm)

b.

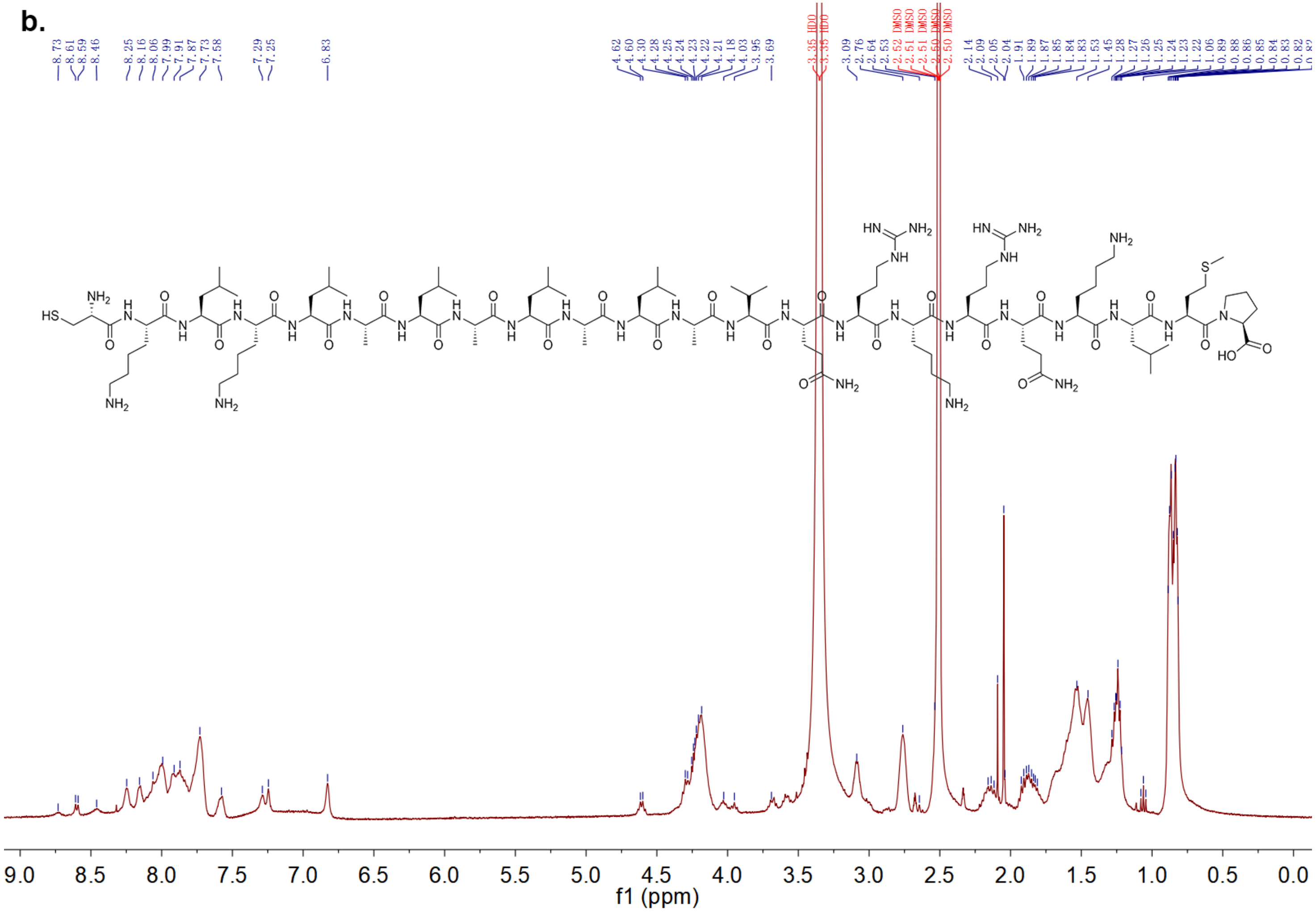

c.

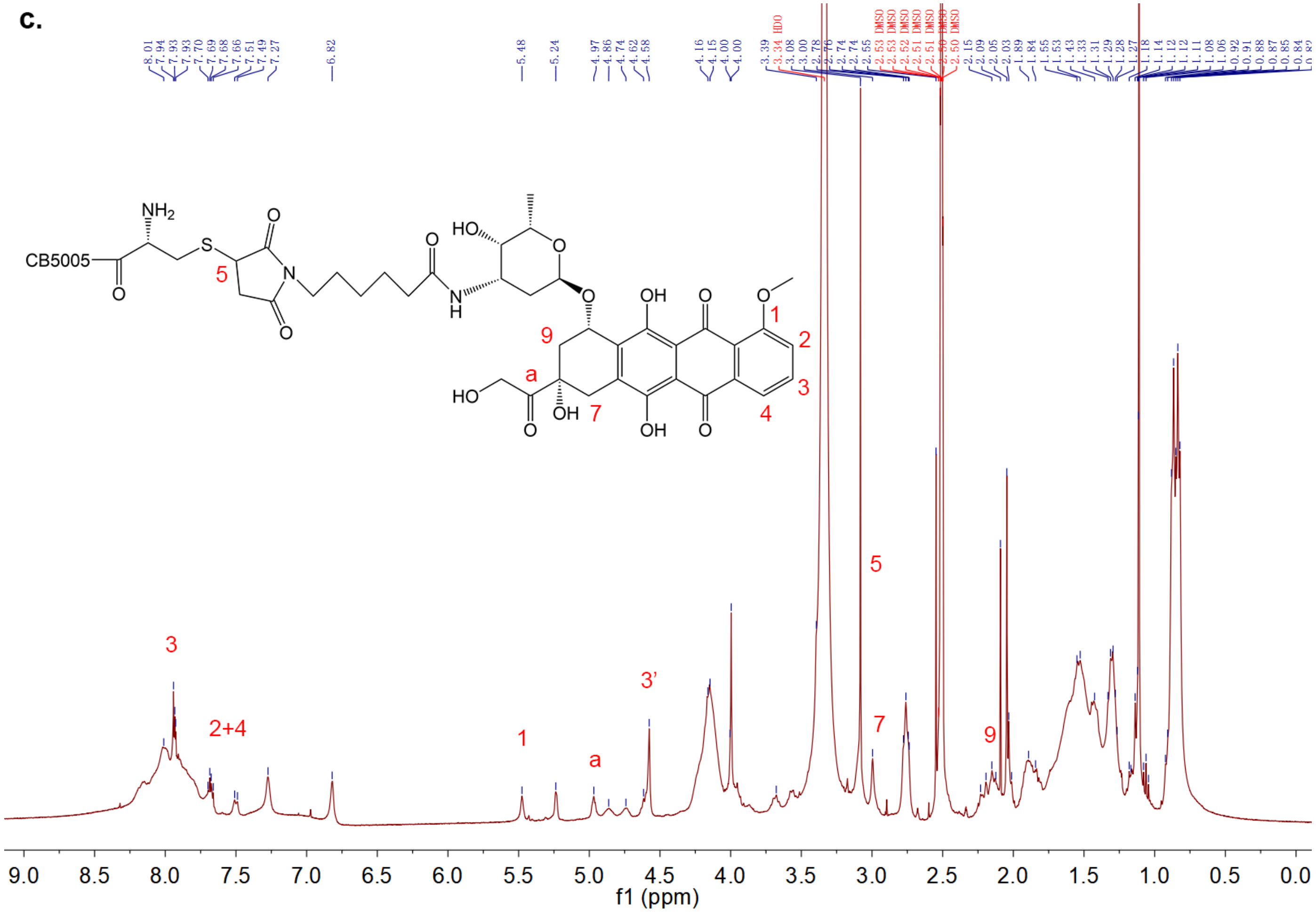

Figure S4. ^{1}H NMR spectrum of DOX (a), CB5005 (b), and [P]DOX (c).

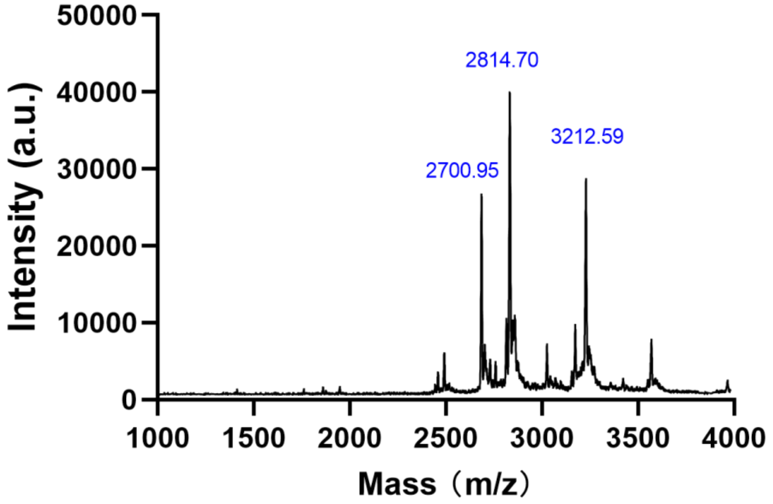

Figure S5. Mass spectrum of ^{P}DOX.

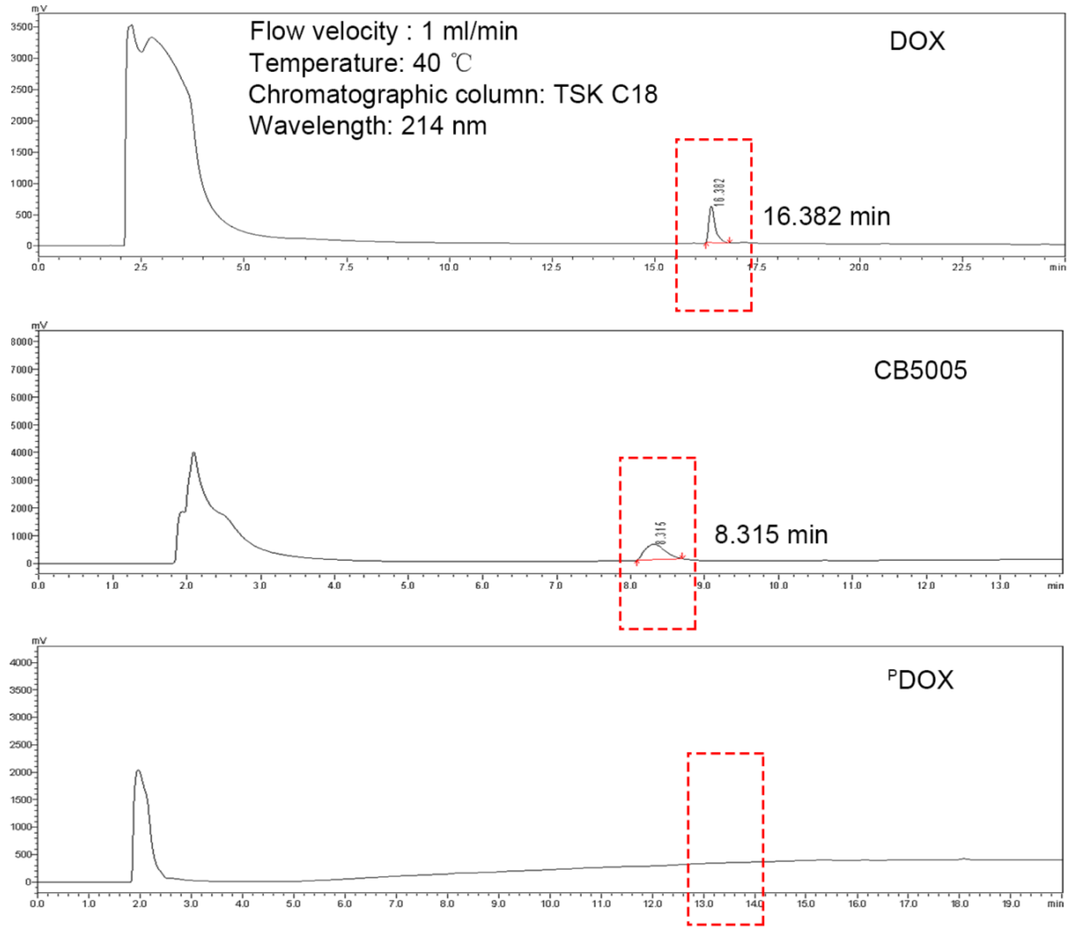

Figure S6. HPLC results of DOX, CB5005, and ^{P}DOX at the wavelength of 214 nm.

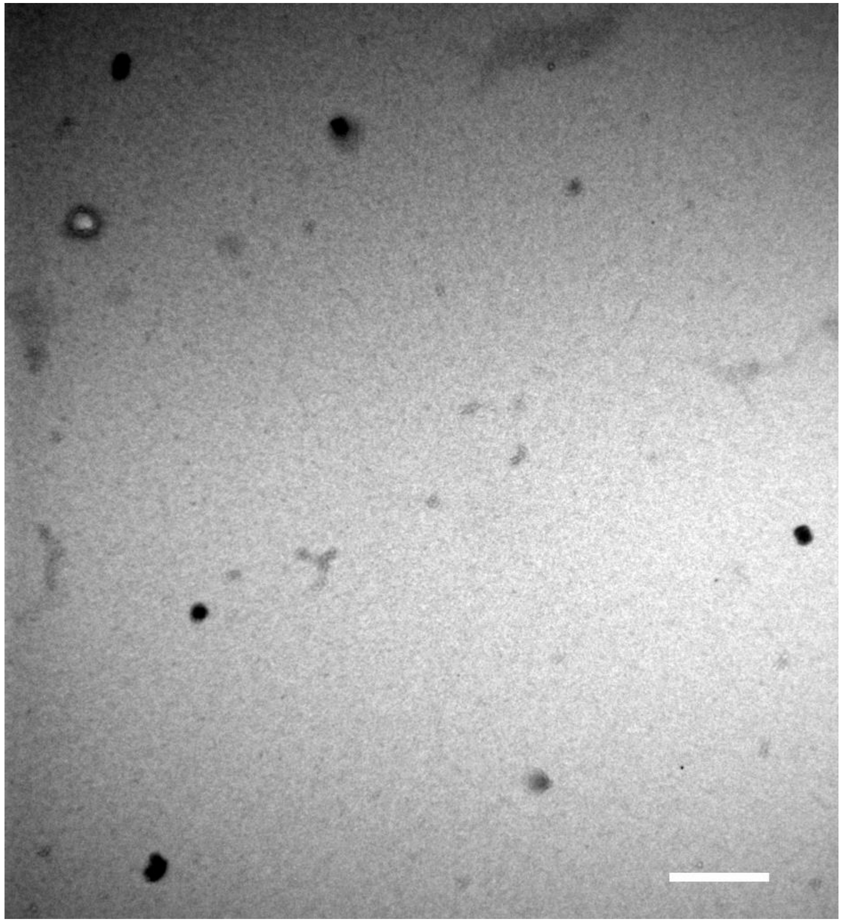

Figure S7. TEM image of Ag-TF@^{P}DOX, the scale bar is 1 μm

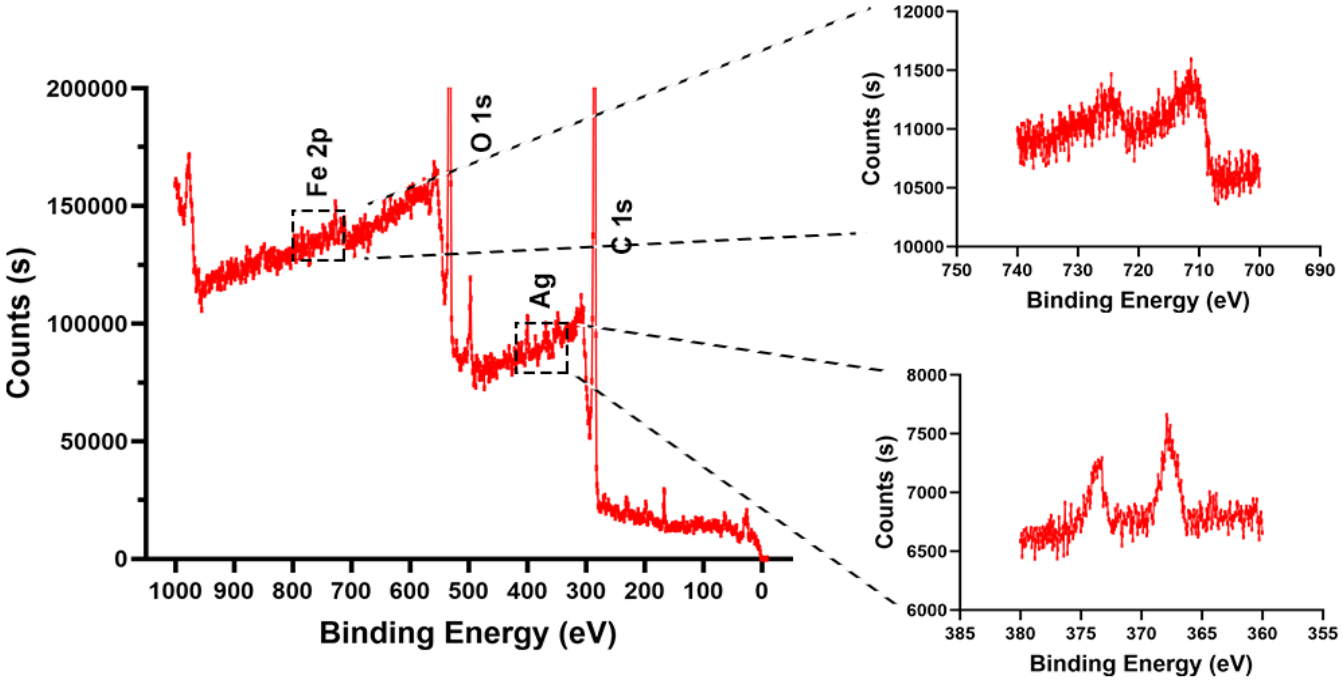

Figure S8. Detailed XPS spectrum of Ag-TF@^{P}DOX in the scanning regions of Ag and Fe.

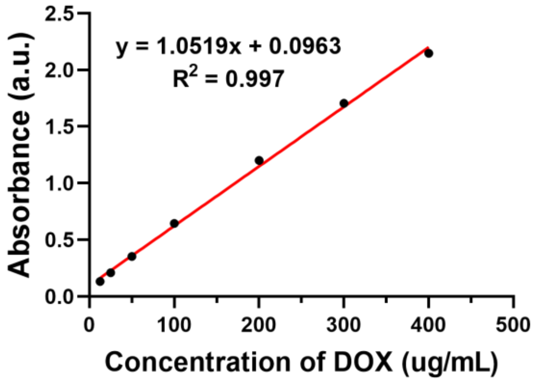

Figure S9. The standard curve of [P]DOX depicted from UV spectrum.

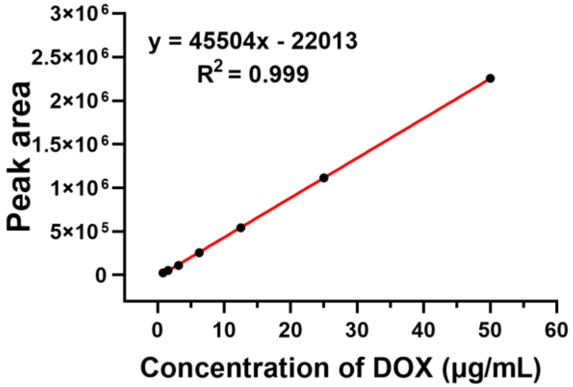

Figure S10. The standard curve of [P]DOX depicted from HPLC measurements.

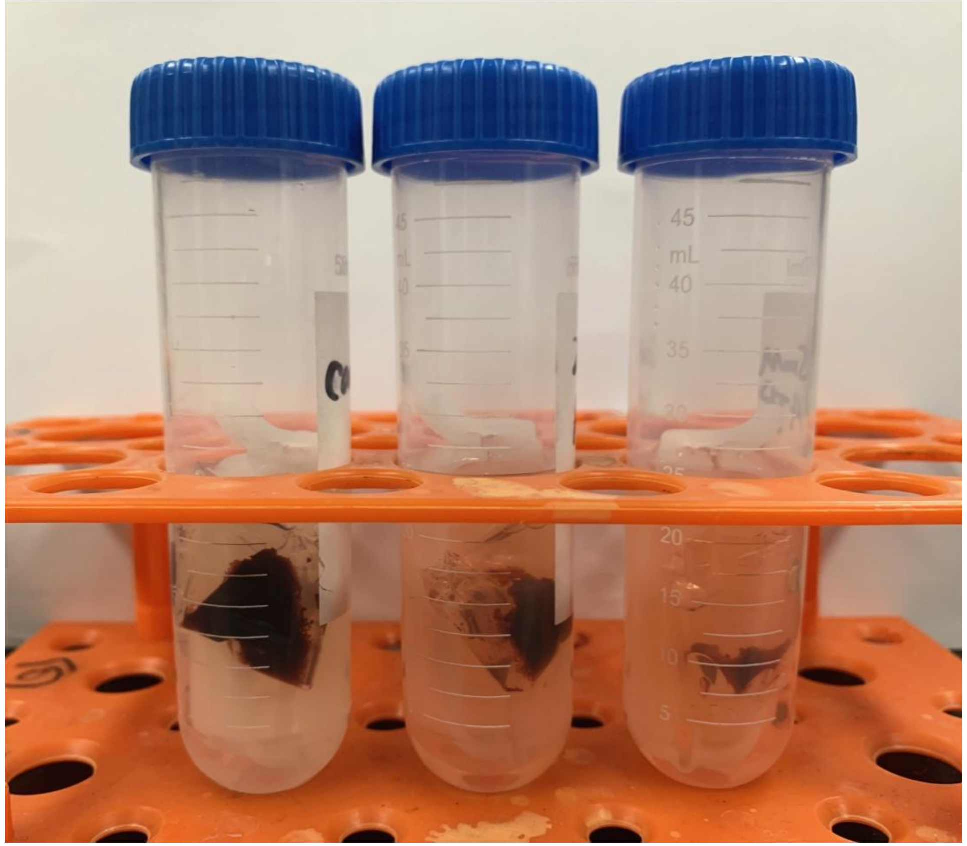

Figure S11. Digital photograph of Ag-TF@^{P}DOX nanocomposites immersed in buffer solution containing ATP concentrations at 0 (left), 2 (middle), or 5 mM (right).

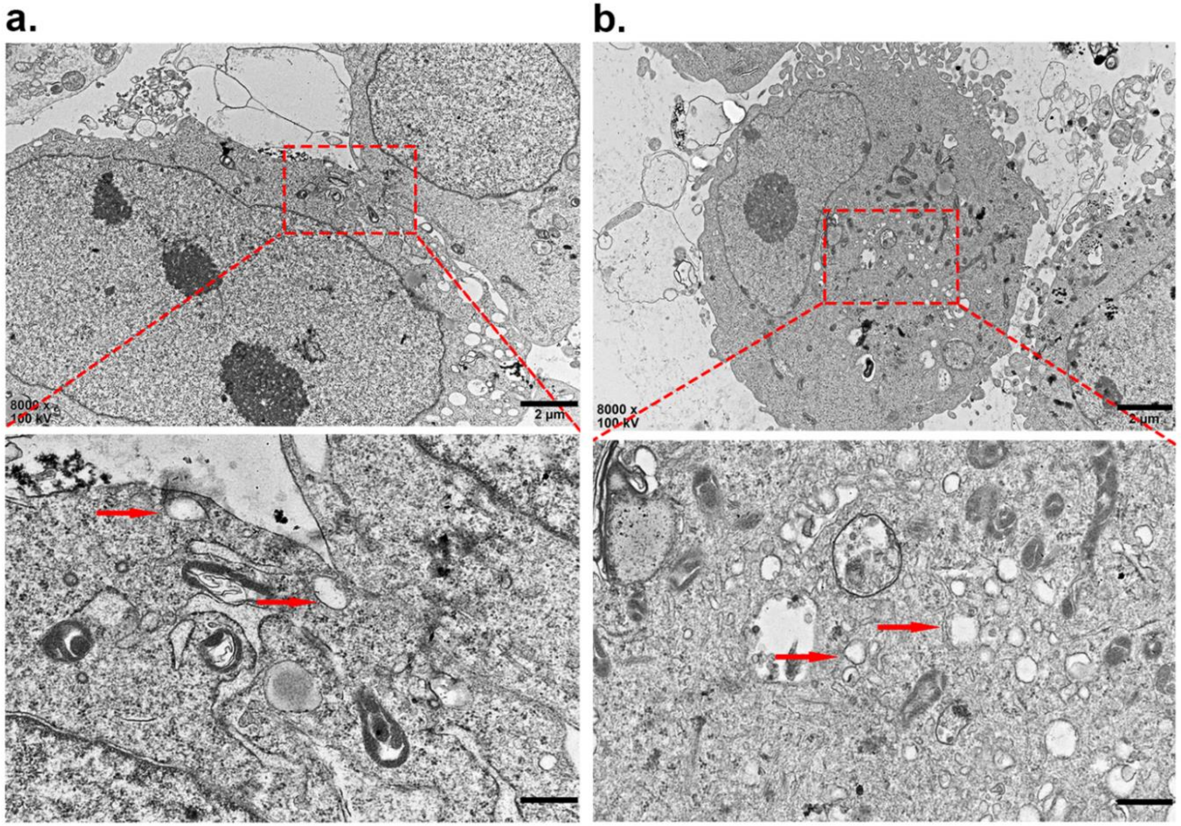

Figure S12. Bio-TEM images of MCF-7/ADR cells incubated with Ag-TF@^{P}DOX for 2 (a) and 4 h (b), the scale bars are 2 μm. The red squares in the images reveal the sites of endoplasmic reticulum in MCF-7/ADR cells, the scale bars are 0.8 μm. The red arrows indicate swollen endoplasmic reticulum.

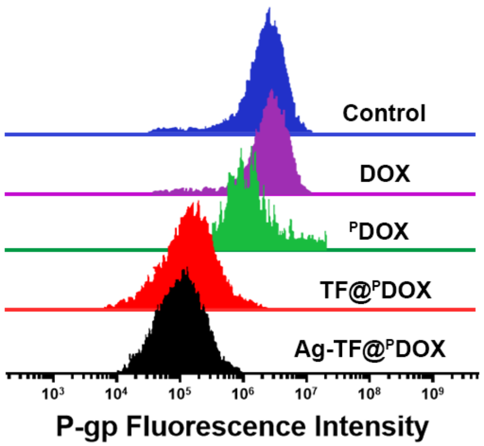

Figure S13. Intracellular P-gp expression quantified by flow-cytometry after different treatments.

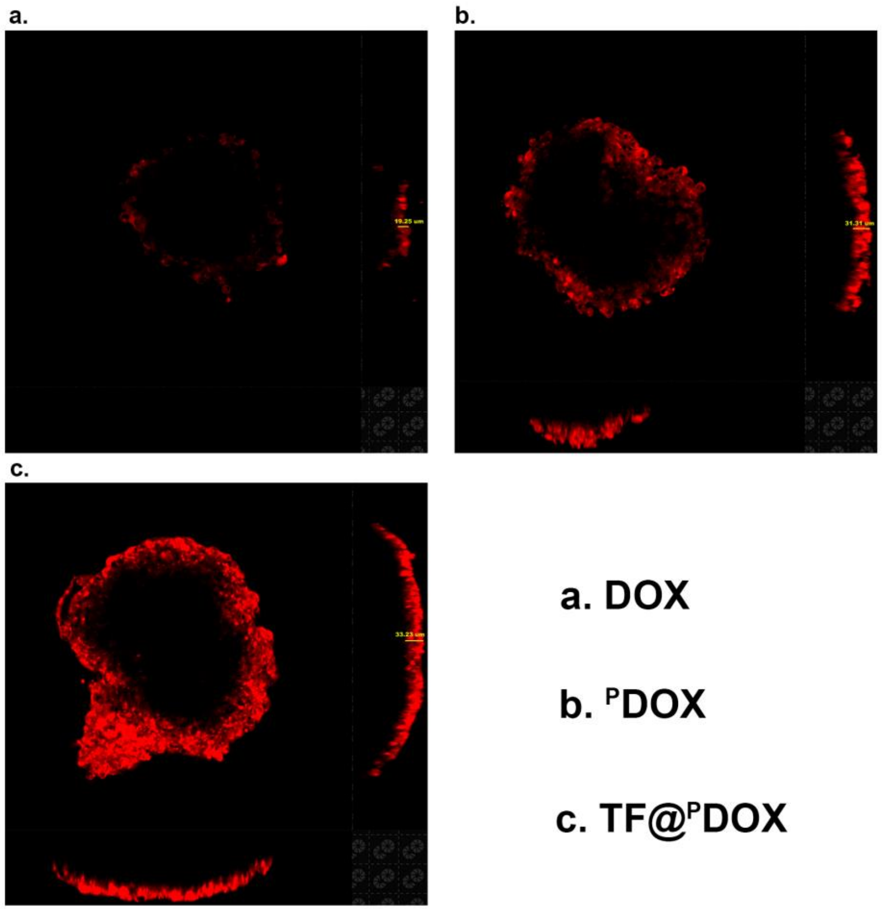

Figure S14. Quantitative analysis of the penetration depth in three-dimensional tumor spheroid models after coincubating with DOX , ^{P}DOX , and TF@^{P}DOX for 6 h.

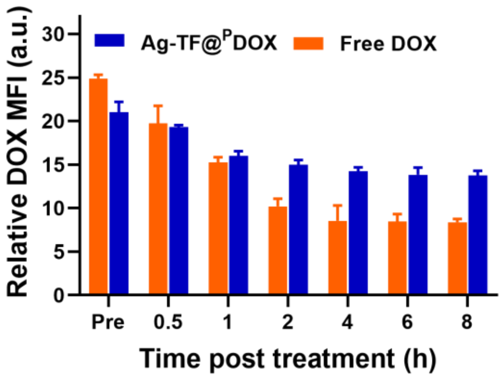

Figure S15. Intracellular DOX fluorescence retention at various time intervals after Ag-TF@^{P}DOX and free DOX treatments, date are presented as mean ± SD (n=3).

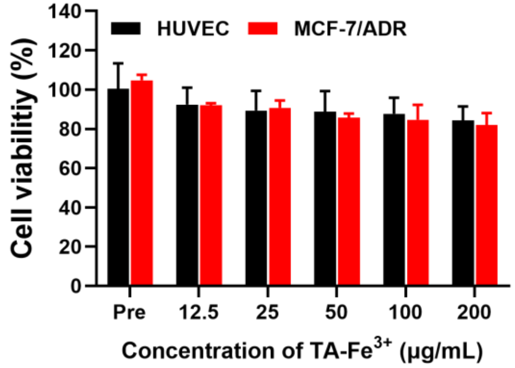

Figure S16. Cell viabilities of HUVEC and MCF-7/ADR cells after incubation with TA-Fe^{3+} at different concentrations, date are presented as mean ± SD (n=3).

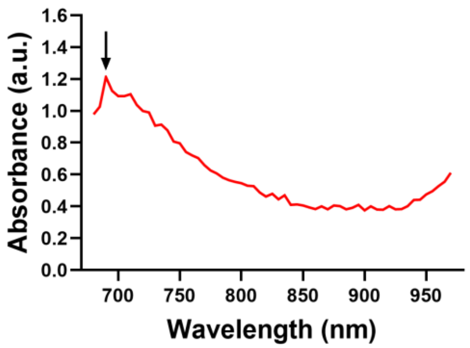

Figure S17. The detection of optimal excitation wavelength by full spectrum scanning from 680 to 970 nm in PAI system.

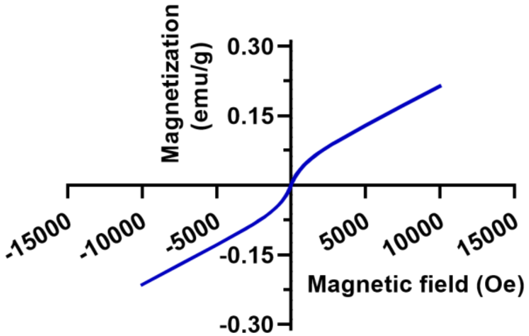

Figure S18. Magnetization hysteresis loop of Ag-TF@^{P}DOX ranging from -10 kOe to +10 kOe at 300 K.

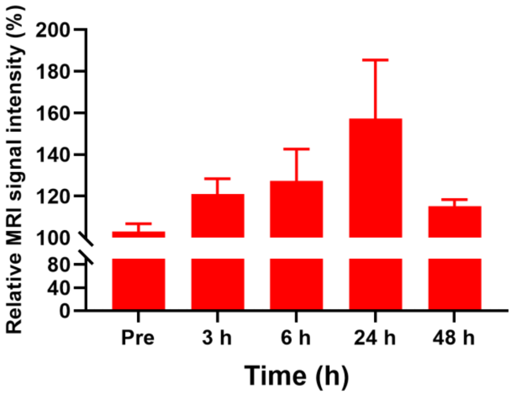

Figure S19. Relative T_1-weighted MRI signal intensity between tumor region and nontumor region after intravenous administration of Ag-TF@^{P}DOX at different time intervials, date are presented as mean ± SD (n=3).

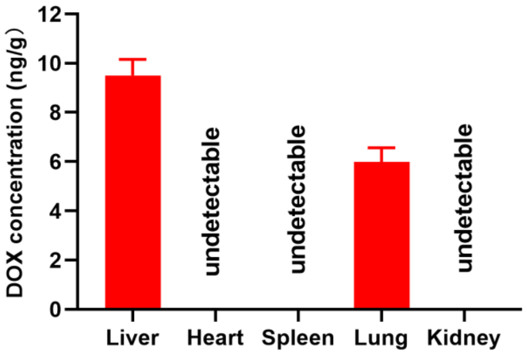

Figure S20. Quantitative biodistribution of DOX in major organs of mice, date are presented as mean ± SD (n=3).

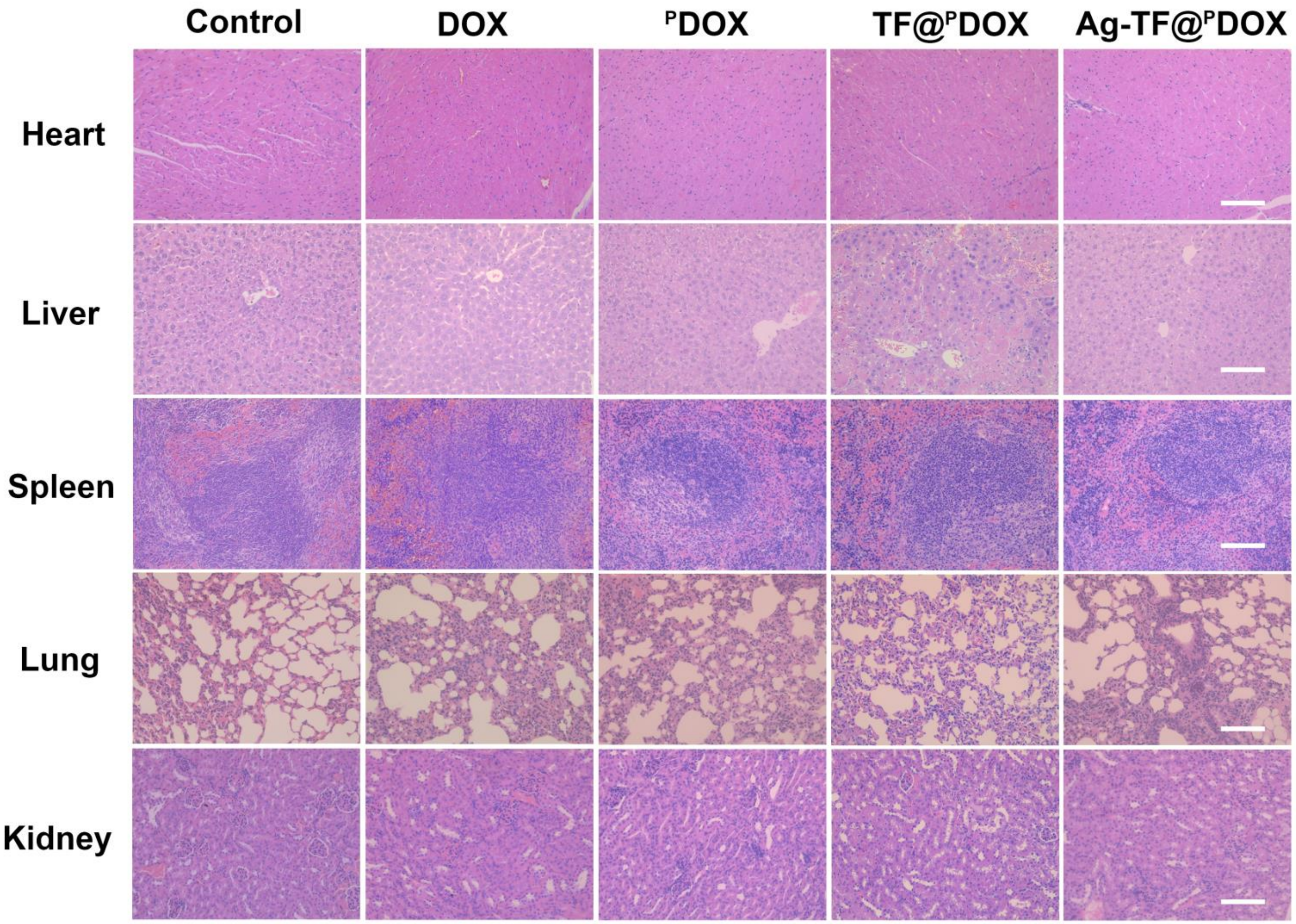

Figure S21. H&E staining of the major organs in mice sacrificed at 14 d after various treatments. The scale bars are 100 μm.

.

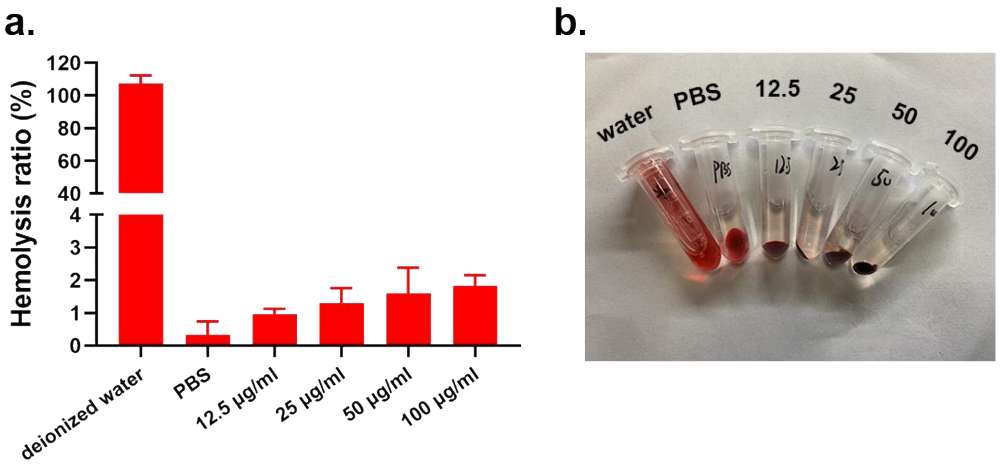

Figure S22. Hemoglobin release from blood cells (a) and hemolysis effect (b) after treating with deionized water, PBS, and Ag-TF@^{P}DOX encapsulating various concentrations of ^{P}DOX (12.5, 25, 50, and 100 μg/ml).

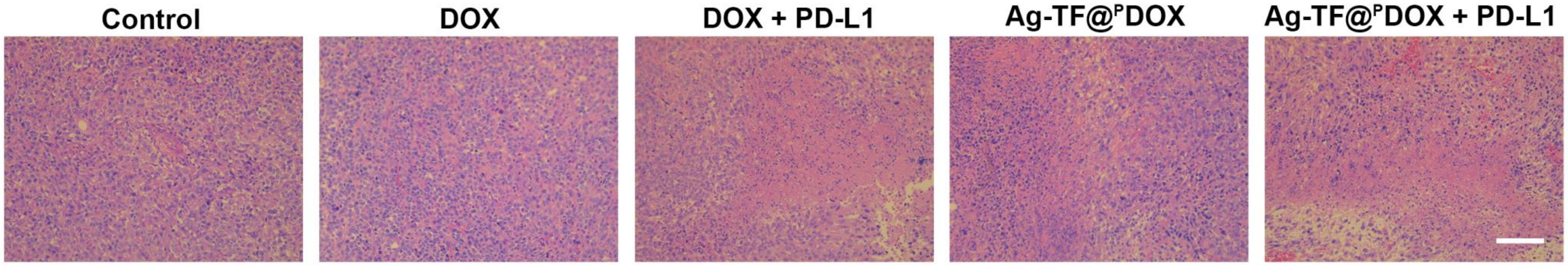

Figure S23. H&E staining on distant tumor sections of 4T1 tumor-bearing mice after various treatments. The scale bar is 50 μm

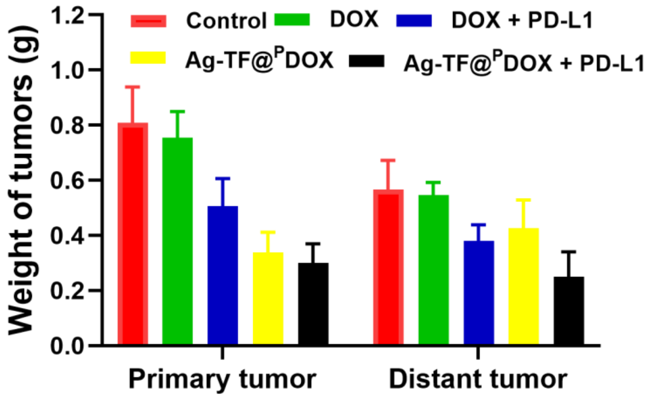

Figure S24. Tumor weight of 4T1 tumors after receiving different treatments, date are presented as mean ± SD (n=5).

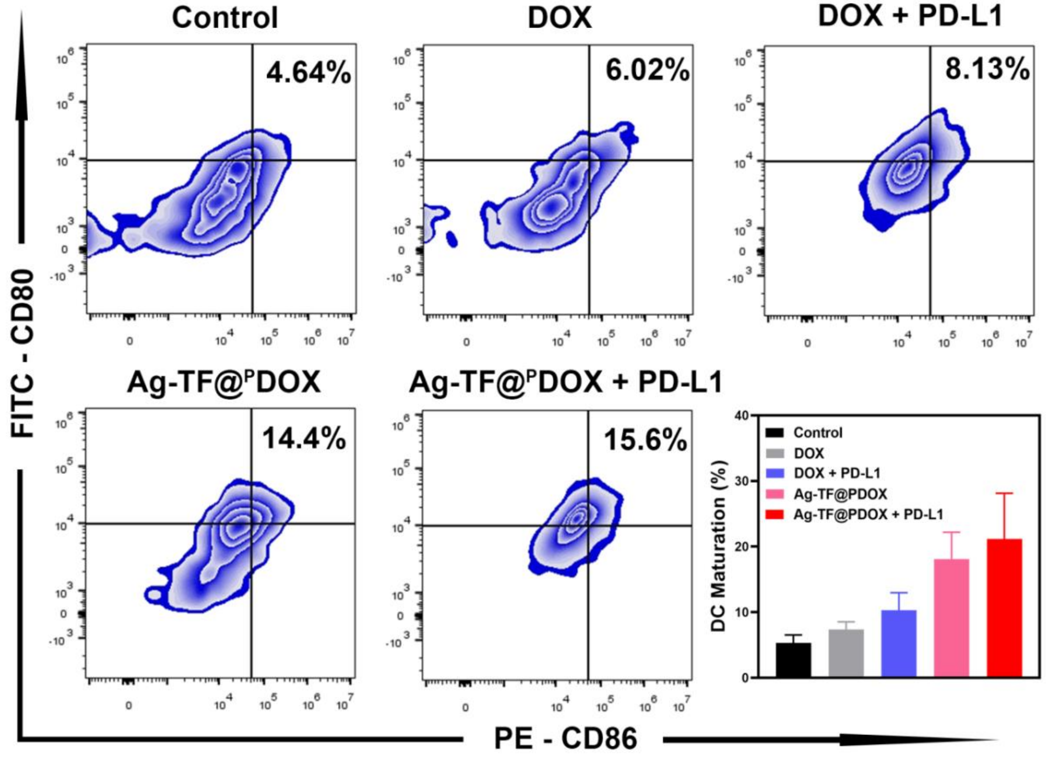

Figure S25. The DC maturation levels in primary tumors of 4T1 tumor-bearing mice the after different treatments, date are presented as mean ± SD (n=3).

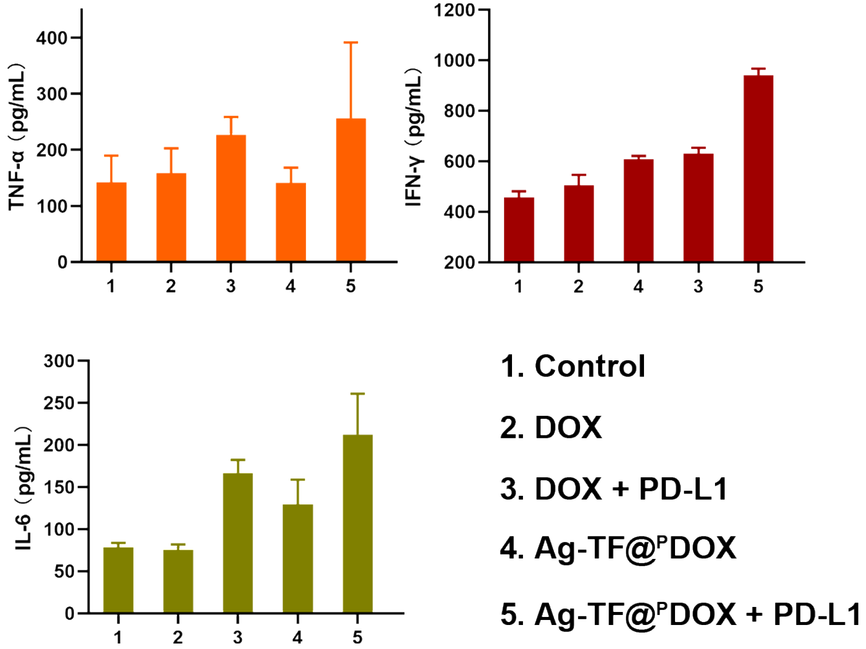

Figure S26. TNF-α, IFN-γ and IL-6 levels in serum from mice of different groups after various treatments, date are presented as mean ± SD (n=3).

Table S1. Entrapment efficiencies, entrapment contents, and size distributions of nanocomposites under different mass ratios of feeding drug : TA- Fe^{3+}.), Date are presented as mean±SD (n=5)

Mass ratio (feeding drug : TA-Fe^{3+})	1:2.5	1:5	1:7.5	1:10
Entrapment efficiency (%)	58.33 ±1.60	79.23 ±2.05	88.33 ±0.83	93.87 ±0.85
Entrapment content (%)	35.90 ±0.95	35.20 ±0.91	30.73 ±0.30	26.80 ±0.20
Size (nm)	117.03 ±3.16	152.16 ±2.05	619.80 ±28.81	1188.86 ±276.65
